# The effect of a multimodal multicomponent Prehabilitation program in Older adults with Chronic limb-threatening Ischemia (POCI-study): A study protocol for a multicenter randomized controlled trial

**DOI:** 10.1371/journal.pone.0354344

**Published:** 2026-07-29

**Authors:** Seline Verbaan, Miriam C. Faes, Elke T. A. M. van Delft, Alexander L. Kooiman, Patrick W. H. E. Vriens, Hidde Jongsma, Ewout W. Steyerberg, Wilbert B. van den Hout, Lijckle van der Laan, on behalf of the POCI-study group

**Affiliations:** 1 Department of Surgery, Amphia Hospital, Breda, the Netherlands; 2 TIAS school for business and society, Tilburg, the Netherlands; 3 Department of Geriatrics, Amphia Hospital, Breda, the Netherlands; 4 Science Department, Amphia Hospital, Breda, the Netherlands; 5 Department of Vascular Surgery, Elisabeth TweeSteden Hospital, Tilburg, the Netherlands; 6 Department of Medical & Clinical Psychology, Tilburg University, the Netherlands; 7 Department of Vascular Surgery, Meander Medical Centre, Amersfoort, the Netherlands; 8 Julius Center for Health Sciences and primary care, University Medical Center Utrecht, Utrecht, the Netherlands; 9 Biomedical Data Sciences, Leiden University Medical Center, Leiden, the Netherlands; National Cerebral and Cardiovascular Center: Kokuritsu Junkankibyo Kenkyu Center, JAPAN

## Abstract

Chronic limb threatening ischemia (CLTI) in older adults is associated with severe morbidity and high mortality. The rising prevalence is largely driven by the aging population and confronts healthcare systems with challenges like increasing demand for chronic care, staff shortage and rising healthcare costs. To improve post-operative outcomes and reduce the burden on healthcare systems, multimodal prehabilitation has gained interest and has the potential to improve post operative outcomes. However, evidence of the effect in older adults with CLTI remains scarce. The aim of this study is to determine whether a multimodal multicomponent prehabilitation program (MMPP) reduces length of stay and improves clinical, patient-reported and economic outcomes of older adults with CLTI. We developed a multicenter randomized controlled trial, with embedded cost-effectiveness analyses.. CLTI-patients aged 65 years or older, planned for revascularization, and their primary informal caregiver (IC) will be eligible. A total of 300 patients will be randomized to receive either standard preoperative care or a 2-week MMPP. All patients receive a general health screening. The MMPP includes physiotherapy and, if indicated, referral to a geriatrician, dietician or smoking-cessation coach, ferric carboxymaltose infusion or pre-arranged homecare. The primary outcome is length of stay. Exploratory secondary outcomes include minor complications, quality of life of patient and IC and health related quality of life. Descriptive secondary outcomes include 30-day and 6-month mortality, major complications, readmissions, burden on the IC, cost-effectiveness; and experiences and preferences regarding shared decision making. To the best of our knowledge, this is the first randomized controlled trial to evaluate the effect of an MMPP within older CLTI-patients. Findings will inform healthcare professionals whether an MMPP should be implemented in routine vascular surgical practice to reduce length of stay and improve clinical and patient-centered outcomes. The study is registered at the International Clinical Trials Registry Platform (NL-OMON58069).

## Introduction

As the population ages, the growing prevalence of chronic limb threatening ischemia (CLTI) has become a major public health concern [1–3], confronting healthcare systems with challenges like increasing demand for chronic care, staff shortage and rising healthcare costs [4,5]. CLTI is the terminal stage of peripheral arterial disease and is characterized by atherosclerotic occlusion of lower-extremity arteries, resulting in ischemic rest pain or tissue loss (ulceration or gangrene), existing for at least two weeks [6–9]. CLTI is often associated with a high hospital (re)admission rate, diminished overall health and quality of life (QoL), places a substantial burden on the informal caregiver (IC) and results in high health-care costs [3,10–12]. Current clinical guidelines recommend early revascularization as primary treatment for CLTI to prevent major limb amputation [8,13]. Despite revascularization efforts, up to one quarter of patients with CLTI remain at risk for major amputation and mortality rates continue to be high, commonly exceeding 50% after 5 years, and increasing with older age [3,14–17]. In addition to patients’ age, the prevalence of co-comorbidities and frailty are likewise associated with higher mortality rates and poorer postoperative outcomes [10,17–21]. The presence of frailty and older age among CLTI-patients is common and the optimal treatment of CLTI-patients therefore requires a multimodal and personalized approach.

To assess the emerging healthcare challenges, the Dutch National Health Care Institute published the ‘Appropriate Care Framework’ [22,23]. Appropriate care in the Netherlands is defined according to four core principles: value-based, patient centered, the right care in the right place and focused on health rather than illness [22]. In line with all four principles of appropriate care, multimodal prehabilitation has emerged as a novel strategy across various surgical procedures with a potential beneficial effect on postoperative outcomes [24–28]. Multimodal prehabilitation aims to enhance patients’ functional capacity and resilience prior to surgery to improve postoperative outcomes and facilitate recovery [29]. Included interventions may consist of physical training, psychological support and nutritional and lifestyle optimization. Studies investigating the effect of mainly exercise prehabilitation in older adults support these earlier reported beneficial effects [30,31]. However, the effect of multimodal prehabilitation specifically in older patients (≥65 years) with CLTI, remains underreported [32]. Therefore, our research group developed a multimodal multicomponent prehabilitation program (MMPP) and demonstrated in an observational cohort study that prehabilitation in older CLTI-patients is safe and has the potential to improve postoperative outcomes [33].

To address another principle of appropriate care, the patient centered approach, shared decision making (SDM) has gained interest. SDM increases patients’ knowledge of treatment options, supports accurate risk perception, improves patient satisfaction and reduces undesired care in certain patient groups [34]. Especially in CLTI-patients, who are often frail and of older age, SDM should be integrated in treatment planning [35]. Although previous research shows increasing attention for SDM in the vascular surgery practice, there may be room for improvements by increasing surgeons’ and patients’ knowledge and awareness on the process of SDM [36–38].

Our previous observational study initiated a paradigm shift in the treatment of CLTI-patients in our own vascular surgery practice, from prioritizing revascularization as soon as possible, to intentionally creating time for prehabilitation. However, implementing prehabilitation in the routine Dutch vascular surgery practice for CLTI-patients has proven challenging. This is possibly due to the fact that evidence is mainly monocenter, observational and lacks a cost-effectiveness analysis, and is not in accordance with current guidelines [8,13]. Furthermore, despite increased attention to SDM in the vascular surgery practice, the process can still be improved and thorough research in this field is lacking.

To advance appropriate care for older adults with CLTI, this POCI-study is designed, the acronym representing a shortened form of the study title: “The effect of a multimodal multicomponent Prehabilitation program in Older adults with Chronic limb-threatening Ischemia.” The POCI-study is a multicenter randomized controlled trial (RCT) evaluating the effect of an MMPP on length of stay (LOS), postoperative complications, hospital readmission and QoL, including cost-effectiveness analyses, in older CLTI-patients planned for revascularization. In addition, the study aims to explore the experiences and preferences of both patients and ICs regarding SDM in the outpatient vascular surgery setting. Lastly, we aim to evaluate the effect of prehabilitation on the burden on the IC of CLTI-patients.

## Methods

### Study design and setting

This study will be a multicenter RCT with embedded cost-effectiveness analyses. The protocol has been developed in accordance with the Standard Protocol Items: Recommendations for Interventional Trials (SPIRIT) guidelines [39] and is registered at the International Clinical Trials Registry Platform (trialsearch.who.int) on July 30^th^ 2025, register number NL-OMON58069. Patients will be randomized in a 1:1 ratio to either the control group receiving the standard of care or the intervention group participating in the MMPP for a minimum of two weeks prior to their revascularization.

The study will be conducted in three non-university large teaching hospitals in the Netherlands: Amphia Hospital Breda, Elisabeth TweeSteden Hospital Tilburg and Meander Medical Center Amersfoort.

### Study status and ethics

Recruitment of participants started in January 2026 and will continue until January 2028. Both patients and their primary ICs will be enrolled over a two-year period and will be followed for six months after revascularization has taken place. The results of the trial will be analyzed within one year after finishing data collection. Data collection is expected to be completed in July 2028.

The study will be conducted in accordance with the principles of the Declaration of Helsinki (64th World Medical Association General Assembly, Brazil, October 2013), the Dutch “Wet Medisch-wetenschappelijk Onderzoek met mensen” (WMO) (“Medical Research Involving Human Subjects Act) and the principles of Good Clinical Practice. This study is approved by the Medical research Ethics Committees United (MEC-U) with registration number R25.080.

### Eligibility criteria

#### Inclusion criteria patient.

Patients of 65 years or older with CLTI who are planned for revascularization are eligible to participate in this study. According to the guidelines of the European Society of Vascular Surgery (ESVS) the diagnosis of CLTI is based on the following criteria [8]:

- Anamnestic complaints of ischemic rest pain or night pain (Rutherford stage 4) OR (Minor) tissue loss, non-healing ulceration or gangrene of any part of the foot (Rutherford stage 5), present for at least two weeks [40].

AND

- Impaired perfusion, quantified by:
  - Absolute ankle pressure < 50 mmHg
  - Absolute toe pressure < 30 mmHg
  - Ankle-brachial index (ABI) < 0.4
  - Flat or barely pulsatile ankle or metatarsal pulse volume recording (PVR) waveforms

#### Exclusion criteria patient.

A potential participant who meets any of the following criteria will be excluded from participation in this study:

- Patients undergoing conservative therapy (including optimal pharmacological treatment of pain, intensive wound care and minor amputations).
- Need for urgent surgery or endovascular therapy within 2 weeks, based on a Wound, Ischemia, foot Infection (WIfI) classification score of either [6]:
  - ‘Wound’ score of W = 3
  - ‘foot Infection’ score of fI = 3
- Patients who are unable to complete questionnaires, due to either lingual or cognitive incompetence.

#### Inclusion criterium informal caregiver.

Adults serving as the primary IC of an enrolled CLTI-patient will be asked to participate in this study. A primary IC is defined as the individual most involved in caring for the CLTI-patient and supporting at least one instrumental activity of daily living of the patient or the contact assisting in treatment-related decisions.

#### Exclusion criterium informal caregiver.

Primary ICs who are unable to complete questionnaires, due to either lingual or cognitive incompetence will be excluded from the study.

### Study outcomes

**Fig 1:**
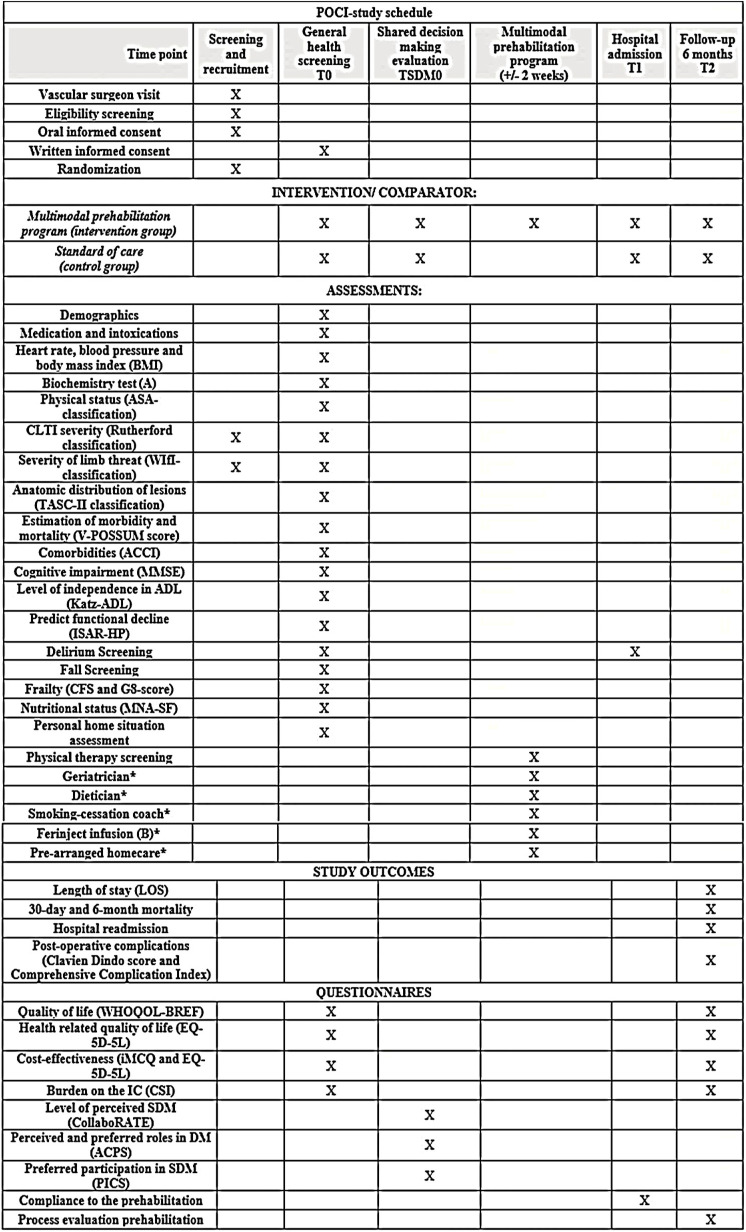
SPIRIT Participant timeline: Schedule of enrollment, interventions, and assessments. All terms marked with * are optional and only provided if indicated; (A) consisting of: hemoglobin, hematocrit, MCV, C-Reactive Protein, leukocytes, thrombocytes, liver enzymes (ASAT, ALAT), electrolytes (sodium, potassium, chloride), albumin, MDRD, urea, creatinine, INR, lipid spectrum including cholesterol, vitamin D, vitamin B12, folium acid, transferrin, ferritin; (B) in case of an iron deficiency anemia, defined by a haemoglobin <8,1 mmol/L for male patients and <7,4 mmol/L for female patients; ASA-classification: American Society of Anesthesiologists Physical Status Classification System; WIfI-classification: Wound, Ischemia, foot Infection classification; TASC-II classification: Trans-Atlantic Inter-Society Consensus II classification; V-POSSUM score: Vascular Physiological and Operative Severity Score for the enUmeration of Mortality and Morbidity; ACCI: Age-adjusted Charlson Comorbidity Index; MMSE: Mini Mental Status Examination; ISAR-HP: Identification of Seniors at Risk – Hospitalized Patients; CFS: Clinical Frailty Score; G8-score: Geriatric-8-score; MNA-SF: Mini Nutritional Assessment short form; SNAQ-RC: Short Nutritional Assessment Questionnaire-score.

An overview of the primary and secondary outcomes is presented in Fig 1.

#### Primary outcome.

The primary outcome of this study is the length of stay (LOS), defined as the number of days a patient is admitted to the hospital after revascularization. If patients are admitted and discharged on the same day, LOS is counted as one day. LOS will be extracted from the electronic patient dossiers.

#### Secondary outcomes.

Secondary outcome measures are divided in four categories:

- Clinical and healthcare related outcomes: postoperative complications, hospital readmission rate, 30-day and 6-month mortality, cost-effectiveness (using the iMTA Medical Consumption Questionnaire (iMCQ) [41] and the EQ-5D-5L questionnaire [42])
- Patient and informal caregiver reported outcomes:
  - Quality of life of both patient and their IC (WHOQoL-BREF) [43]
  - Health related quality of life of patients (EQ-5D-5L) [42]
  - Burden on the IC (CSI) [44]
- Shared decision making
  - The level of perceived SDM of the patient and their IC (CollaboRATE) [45]
  - Perceived and preferred roles in decision making (DM) of the patient and their IC (ACPS) [46]
  - Preferred participation in SDM of the patient and their IC (PICS) [47]
- Study specific outcomes in the intervention group: compliance to the prehabilitation program and process evaluation of the prehabilitation program after 6 months, using effect evaluation forms designed by the research group, see ‘S3 File. Process evaluation questionnaires POCI-study’.

Of all secondary outcomes, only minor complications (Clavien Dindo classification I and II), quality of life and health related quality of life are confirmatory outcomes. All other secondary outcomes require bigger sample sizes to detect a difference with reasonable power and are therefore exploratory endpoints (all preliminary calculations were conducted from https://sample-size.net)

### Study procedure

**Fig 2:**
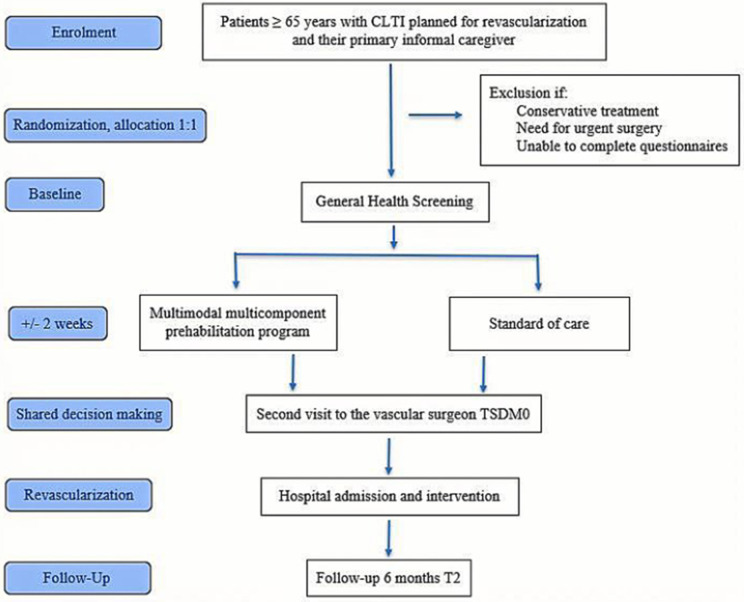
Flow diagram POCI-study.

The SPIRIT participant timeline and study flow diagram are presented in Figs 1 and 2.

#### Participant screening and recruitment.

In all three participating centers, patients of 65 years or older with suspected CLTI, will be examined on the outpatient clinic by a vascular surgeon. Confirmation of the diagnosis is based on the ESVS guidelines [8]. The severity of CLTI at initial presentation is scored by using the Rutherford and WIfI-classification [6]. Additional imaging is requested by the vascular surgeon and may be performed by duplex, Magnetic Resonance Angiography or Computed Tomography Angiogram. Vascular pathology will be scored using the Trans-Atlantic Inter-Society Consensus Document on Management of Peripheral Arterial Disease II (TASC-II) and description of the crural outflow in 1, 2 or 3 arteries [7,9,48]. All patients with newly diagnosed CLTI, who are likely to require surgical or endovascular revascularization and who meet the eligibility criteria, are invited to participate in the study. Patients’ primary IC will also be invited to participate in the study. Patients remain eligible, even if their primary IC declines participation. Primary ICs can only be included in combination with a patient and are automatically assigned to the same study group as the patient. Written and verbal study information is provided by the vascular surgeon, a researcher or a vascular nurse practitioner (VNP). Questions will be answered and potential participants are asked if they are willing to participate in the study. Patients and their IC are offered sufficient time to process the study information and consider participation. If needed, telephone contact is made by the researcher or VNP the day after the outpatient clinic visit.

#### Informed consent and randomization.

Patients who agree to participate, provide oral informed consent, after which they are immediately randomized to the prehabilitation group or the control group. Enrollment of the patient and their IC occurs on the day of, or the day after, the first outpatient clinic visit. This ensures that prehabilitation starts within a few days, parallel to the treatment planning, without delaying the moment of revascularization. All participants will be scheduled within a few days for an appointment with the VNP or researcher for a general health screening (T0), during which written informed consent will be obtained.

Participants are randomized to either the prehabilitation group or the control group using a variable block randomization (2:4:6) procedure to minimize the risk of unequal group sizes and to reduce predictability of assignments. To further ensure comparability between study arms, randomization is stratified by the participating hospital, to eliminate systematic imbalances between hospitals and enhance internal validity. The randomization procedure will be carried out using the validated electronic data capture system Castor EDC. This study is not subject to blinding due to the extensive and active nature of the intervention.

#### General health screening (T0).

All included patients undergo a general health screening by a trained VNP or researcher as part of the standard care. The content and details of this assessment are shown in Fig 1. Patients are requested to bring their primary informal caregiver, to optimize the (hetero)anamnesis, to ensure understanding of the provided information, and assess the availability of informal support. At the end of the visit all patients and informal caregivers are asked to complete the baseline questionnaires (Fig 1). After completing the questionnaires, patients in the control group conclude their appointment. Patients randomized to the intervention group proceed with the MMPP.

#### Study intervention–Multimodal multicomponent prehabilitation program.

The MMPP has a minimum duration of two weeks, considering both the expected time needed to achieve clinically meaningful results and the time in which treatment is planned and must take place [33]. Patients’ IC will be an active participant in the MMPP, as they can supervise physical training at home, help patients with following advices and can participate in decision making. The program consists of six components: a mandatory visit to a physiotherapist, and, if indicated, referral to a dietician, geriatrician or a smoking-cessation coach; ferric carboxymaltose infusion or pre-arranged homecare. In addition, to reduce the burden on patients and their IC, all required appointments are combined into a single morning or afternoon if possible. Specific indications and details of the components of the MMPP are shown in Table 1. At the start of the visit to the MMPP, all patients receive an information brochure of the prehabilitation outpatient clinic, containing general information of the outpatient clinic, the physiotherapy handbook and the dietetics information brochure (see S4. File Information brochure outpatient clinic).

I. Physiotherapist

Immediately after the general health screening, patients in the intervention group are screened by a physiotherapist. Patients’ physical state, muscle strength and cardiopulmonary condition are examined by conducting several tests, as described in Table 1. All tests are widely used and chosen taking into account the pain-limited performance capacity of most CLTI-patients. The TUG-test and grip strength are performed in agreement with the geriatricians to give an indication of patients’ level of frailty [49]. Based on the results of the tests, patients are provided with a personalized, home-based training schedule, targeting muscle strength, cardiopulmonary condition and improving patients’ ability to conduct transfers. To ensure uniformity of the provided screening, advices and the amount and intensity of the prescribed exercises in all participating centers, the physiotherapy handbook was designed (S4. File Information brochure outpatient clinic). To increase compliance, patients are provided with a diary to track their daily activities. Their IC is instructed to support completing the exercises and to help keep track of patients’ compliance in their diaries.

**Table 1:** Indications and details of the components of the MMPP.

Component	Indications	Tests	Interventions
Physiotherapist	All patients in the intervention group	Maximal inspiratory pressure (MIP) The ‘De Morton Mobility Index’ (DEMMI) Grip strength Timed Up and Go test (TUG) Timed Chair-Stand test (TCS) 10 Meter walking test	Home-based exercises
Geriatrician*	- MMSE-score < 26 - TUG-test > 14 seconds - Previous delirium - Polypharmacy > 4 (cardiovascular risk management medication not included) - Alcohol consumption > 7 units/week - G8-score < 14	Comprehensive Geriatric Assessment (CGA)	Preventive measures for functional decline and delirium Prescribe supplementary vitamins*
Dietician*	- SNAQ-RC score > 3 - Low protein intake - Unintentional weight loss	Body Mass Index (BMI) Mini Nutritional Assessment short form (MNA-SF) Short Nutritional Assessment Questionnaire-score (SNAQ-RC)	Dietary advice High protein nutrient drinks*
Smoking-cessation coach*	Active smokers		Individualized or group-based guidance
Ferric carboxymaltose infusion*	- Hemoglobin < 8,1 mmol/L for male patients - Hemoglobin < 7,4 mmol/L for female patients		1000mg intravenous ferric carboxymaltose infusion
Pre-arranged homecare*	Expected problems after discharge or caregiver burden		Pre-arranged homecare

All components marked with * are optional and only provided if indicated.

II. Geriatrician

If indicated (see Table 1) patients are referred to a geriatrician. Patients undergo a comprehensive geriatric assessment (CGA) to assess frailty and the risk of developing a delirium. Previous studies showed that performing a preoperative CGA in older vascular surgery patients is associated with shorter LOS and lower complication rates [50,51]. Based on CGA findings, the geriatrician provides tailored information to patients and their IC, including preventive measures for functional decline and delirium after revascularization. Furthermore, if indicated based on the results of the biochemistry tests, patients are prescribed supplementary vitamins.

III. Dietician

If indicated (see Table 1), the dietician assesses patients’ nutritional status. All patients are provided with oral and written information on preoperative optimization of their diet, mainly focused on a sufficient protein intake (see S4 File Information brochure outpatient clinic). Protein targets are calculated as 1,2g/kg body weight, taking into account patients’ BMI and renal function and adjusted accordingly if necessary. If patients reach only 50–75% of their protein target, they initially obtain recommendations on a high protein diet. In case those recommendations are insufficient, oral nutritional supplements are prescribed and patients receive a control appointment with the dietician.

IV. Smoking-cessation coach

Active smokers are offered a referral to a smoking-cessation coach to receive guidance in reducing nicotine abuse. The coach intends to see patients within three days after referral and provides individualized or group-based guidance, depending on patients’ preferences.

V. Ferric carboxymaltose infusion

In the presence of anemia, patients receive a dose of 1000 mg intravenous ferric carboxymaltose in 15 minutes, administered during a day-care admission. If hemoglobin concentration is below 6.2, patients are referred to internal medicine for further evaluation of possible underlying diseases. A previous study evaluating the effect of ferric carboxymaltose infusion as part of a prehabilitation program for older surgical patients, demonstrated the administration is safe and eliminates differences in hemoglobin concentration at discharge between patients who initially presented with anemia and those without anemia, regardless of iron concentration at initial presentation [52].

VI. Pre-arranged homecare

If problems are expected in patients’ home situation after discharge from the hospital, for example due to functional decline after revascularization or an overburdened IC, pre-arranged homecare is initiated by the VNP or researcher.

#### Multidisciplinary meeting.

All patients are discussed in a weekly, multidisciplinary meeting with vascular surgeons and interventional radiologists. This session results in a treatment advice for either surgical or endovascular revascularization, based on the type of pathology, the imaging findings and the results of the general health screening. If conservative treatment is proposed, or patients require primary major amputation, they are excluded from this study.

#### Second visit to the vascular surgeon (TSDM0).

The vascular surgeon will discuss the proposed treatment plan with the patient and their IC in a second outpatient clinic visit. At the end of the visit, both patient and their IC are asked to complete the questionnaires regarding the SDM process (Fig 1). Together with the questionnaires, explicit instructions are provided to complete the questions solely based on their experiences with the visit to the vascular surgeon.

#### Hospital admission (T1).

At hospital admission, approximately two weeks after inclusion, patients in the intervention group complete a process evaluation form to assess adherence and experiences with the prehabilitation program (S3 File. Process evaluation questionnaires POCI-study). Patients’ diaries serve as a reference throughout this evaluation.

#### Follow up 6 months (T2).

All patients receive postoperative follow-up, according to the local hospital guidelines. At six months after revascularization, patients and ICs are invited by post to complete the same questionnaires they completed during the general health screening. In addition, participants in the intervention group are asked to complete another process evaluation form, evaluating their experiences and preferences with the MMPP on the long-term (S3 File. Process evaluation questionnaires POCI-study).

### Patient and informal caregiver involvement

The development of this study has been supported by the Dutch senior citizens associations “Katholieke Bond van Ouderen” and “Protestants Christelijke Ouderenbond” (KBO-PCOB) (“Catholic association of elderly people” and “Protestant Christian seniors association”) and the patient council of the Amphia Hospital. We incorporated the advises and recommendations from the process evaluation forms from our previous observational study [33] and a previous pilot interview study in CLTI-patients and their ICs [11], in the information brochures, patient information folders and the shared decision-making questionnaires of the POCI-study Approximately three months after study initiation, halfway through the study and at study completion, the MMPP information brochures will again be evaluated, together with patients and ICs and revised as needed.

### Statistical analysis

#### Sample size.

Sample size was calculated for the primary outcome LOS. Previous observational data showed that prehabilitation was associated with a difference of 2 versus over 4 days of hospital stay in the prehabilitation group compared to the control group [33]. A halving in length of stay requires 54 patients in total (27 per group, assuming a SD of log (LOS) of 0.9, as in the previous study). With a total of 300 patients, 100 from each participating center, the study has 80% statistical power with 2-sided alpha set at 5% to detect a 25% reduction (factor 0.75) in LOS. Sample size calculation was done using the online sample size calculators at https://sample-size.net/.

#### Statistical analyses.

Statistical analyses will be performed using IBM SPSS statistical software (SPSS Inc., Chicago, Illinois, USA). Results of categorical data variables are described in frequencies with percentages and differences will be tested using the Chi-square test. Ordinal variables will be tested using the Mann-Whitney U-test. Adjusted analysis will be performed using regression analysis with prognostic baseline characteristics (e.g., gender, age). Continuous data are described as median (interquartile range) and Mann-Whitney U-tests will be performed to test for group differences. Continuous outcomes are analyzed using a linear regression model, whereas binary outcomes are analyzed by using a logistic regression model. To assess the primary study parameter LOS, a linear regression model including important covariates (such as age, gender, surgical history, comorbidities, use of medication, smoking status, use of alcohol, home situation) that may contribute to the outcome of LOS will be used. LOS is expected to be skewed; therefore, a log-transformation will be performed. Hospital site will be a stratification factor in the regression analysis. Repeated measures will be analyzed with mixed effect models with random effects per patient to address patient clustering. Missing data will be studied for specific patterns of occurrence, and multiply imputed. The primary data analysis will be done according to the intention-to-treat concept. Secondary, a per protocol analysis will be performed.

We conducted power calculations for the secondary outcome’s minor complications, quality of life and health related quality of life at https://sample-size.net. In the previous study by Meulenbroek et al. [33] prehabilitation was associated with a difference in minor complications of 12% (26% vs 14%). This difference would require a sample size of 346. With 300 patients we will have reasonable power (69%) for detecting a difference in minor complications.

Quality of life (WHOQOL-BREF) and health related quality of life (EQ-5D-5L) will be evaluated at baseline (pre-intervention) and at 6 months post-operatively. With a total sample size of 300, this study has a statistical power of 80% (two-sided, α = 0.05) to detect a standardized Effect Size (Cohen’s d) of 0.36 or larger on the domains of the WHOQOL-BREF, the EQ-5D index score and the EQ-VAS. Therefore, the sample size is considered adequate to detect small-to-medium clinical effects. Interpretation of the results will focus on the Minimal Clinically Important Difference (MCID). The exact MCID for both the WHOQOL-BREF and the EQ-5D-5L scores in CLTI patients has not been decided yet. Therefore, based on an earlier systematic review by Norman et al. [9] we will consider an effect size of 0.5 SD as clinically relevant.

For the outcomes with limited power for comparative analyses, we will provide point estimates with 95% confidence intervals to caution against overinterpretation of negative findings as proof of equivalence.

#### Interim analysis.

Interim analysis shall be conducted for the meetings of the data safety monitoring board (DSMB) and will focus on monitoring complication rates. Data will be presented descriptively as percentages for each group.

#### Cost-effectiveness analyses.

A comprehensive economic evaluation will be performed, including a cost analysis from hospital perspective, a cost-utility analysis from societal perspective (CUA, i.e., costs per QALY), and a budget impact analysis (BIA). In all three analyses, care with and without prehabilitation will be compared according to intention to treat.

- For the hospital cost analysis, six-month hospital costs, including interventions and stay, will be assessed from the financial records of all three participating centers.
- For the CUA, costs from a societal perspective will be compared. Costs outside the hospital such as wound care, physiotherapy, home care and informal care will be assessed using a shortened version of the iMCQ questionnaire at six months [41]. Care will be valued according to Dutch reference prices, including travel costs. Productivity costs are excluded, because of the low labor participation in this 65 + study population. QALYs will be calculated using the Dutch tariff for the five-level EuroQoL EQ-5D (assessed at 0, and 6 months), with a sensitivity analysis using the EuroQol visual analogue scale [42]. Incremental average six months costs and QALYs will be compared using net-benefit analysis with multiple imputation to account for missing data.
- The BIA will estimate the financial impact of different implementation scenarios at the national level. The analysis will be conducted from the perspectives of society, hospital and insurers, using the ZonMw BIA guideline and tool [53]. The BIA will be based on the costs as estimated during the study, and the expected numbers of patients in the Netherlands. Healthcare will be valued according to cost prices (for the societal perspective) or NZa prices (for the hospital and insurer perspectives). Costs will be estimated per 1-year budget period for a time horizon of five years, assuming 50% to 100% implementation after four years.

### Safety considerations

The intervention used in this study has previously demonstrated to be safe in CLTI-patients [33]. During the study, each participating center is responsible for reporting all adverse events to the local principal investigator. Severe adverse events are reported to the MEC-U and the DSMB. Patients will still be included in the database and will not be withdrawn from this study in case an adverse event occurs.

#### Data safety monitoring board (DSMB).

An independent DSMB will be established for this trial. None of the DSMB members are involved in the study in any other capacity.. The DSMB will monitor participant safety throughout the trial, primarily based on the results of the interim-analyses. The DSMB is responsible for assessing whether the MMPP continues to meet safety standards and for issuing recommendations regarding continuation, modification or early termination of the study. Alle recommendations by the DSMB will be communicated to the study sponsor and the principal investigators of all participating centers. The DSMB may recommend premature termination of the study if any notable negative effects emerge in the intervention group, compared to the control group.

### Data management plan

#### Data handling and storage.

Data will be handled confidentially. Clinical data will be collected from the electronic medical records and questionnaires by the PhD student or researchers. Completed questionnaires and informed consent forms will be securely stored in locked cabinets at each participating site, accessible only by authorized personnel. A dedicated digital study folder will be created in each participating hospital in which the digital data is saved. Organization and storage of de-identified data and questionnaires will be managed using Castor SMS and Castor EDC, a clinical data management platform. Data handling procedures adhere to the Dutch “Uivoeringswet AVG, UAVG” (Act on Implementation of the General Data Protection Regulation).

Participants may withdraw from the study at any time after providing consent. Withdrawn subjects will not be replaced and data collected prior to withdrawal will remain part of the study dataset. Routinely collected data regarding LOS, mortality, hospital readmission rate and complications, will continue to be extracted from patients’ electronic medical records.

#### Data monitoring.

Monitoring of informed consent and study data will be performed by the project leader, principal investigators and designated researchers at the participating centers. An independent data monitor will conduct annual site visits to ensure data quality, accuracy and protocol adherence. During these visits information will be reviewed regarding the date of inclusion of the first participant, number of participants included and number of participants that have completed the trial, serious adverse events, other relevant issues or deviations, and protocol modifications.

### Dissemination plan

The main results of this study will be disseminated through publication in an open access peer-reviewed journal. Additional findings will also be disseminated in subsequent publications and presented at national and international scientific conferences. To reach the broader public, results will also be communicated through mainstream media channels in clear, accessible language.

## Discussion

To the best of our knowledge, this study is the first RCT with embedded cost-effectiveness analyses that will investigate the effect of a multimodal multicomponent prehabilitation program on LOS in older adults with CLTI. Earlier studies suggesting a beneficial effect of multimodal prehabilitation included patients planned for various types of surgery, described heterogeneity in prehabilitation programs and did not specifically focus on older adults [24–28]. Studies investigating the effect of prehabilitation specifically in older adults are scarce and mainly focus on exercise prehabilitation [30,31,54]. Due to the high prevalence of frailty among older CLTI-patients [20], geriatric co-management might be beneficial, whereas the presence of frailty in vascular surgery patients has been associated with higher mortality and complication rates [10,19–21]. Two recently published studies in older (≥65 years) vascular surgery patients, showed a reduction of LOS and complication rate in patients who were referred to a geriatrician prior to their intervention [50,51]. The typical CLTI-patient is old and frail, has multiple comorbidities and often has a history of tobacco use, and therefore, our study team strongly believes in the power of the multicomponent, multimodal and personalized aspect of our prehabilitation program [33].

There are several strengths incorporated in the design of this study. Firstly, this will be a randomized controlled trial conducted in three non-university top-clinical hospitals in the Netherlands, ensuring a high internal validity and reducing the risk of confounding. Secondly, to assess frailty, the clinical frailty scale (CFS) is used [55]. This is a widely used, user-friendly scale, with a good predictive ability on adverse events. This scale has already been proven useful in vascular surgery and by using this scale, results of this trial might be compared to results of previous and future research [56]. Lastly, the MMPP is primarily home-based and will only require a single hospital visit. Although a recent randomized controlled trial showed no favorable effect of home-based multimodal prehabilitation on patient-centered outcomes or postoperative complication rates in older surgical patients [57], we aim to reduce the burden of participation on our population of older CLTI-patients and their IC.

There are some limitations inherent in the design of this study. Firstly, due to the study design, blinding of health-care workers and researchers is not possible. This may introduce the risk of researcher bias or detection bias. Data extraction will be conducted by the PhD-student or researchers at each participating site. All will not be blinded to group allocation, due to their active role in the recruitment of patients and their responsibility to conduct the general health screening of study participants. While blinding is not feasible, objective outcome measures and standardized assessment procedures will be used to minimize potential observer bias. In addition, due to the active nature of the intervention, masking of participants is not feasible, which potentially risks information bias in patient reported outcomes or performance bias in the control group. Secondly, severe cognitive impairment, to the extent patients are not able to complete the questionnaires, is an exclusion criterium of this study. We are aware, the incidence of cognitive impairment in our frail CLTI-population might be high [58]. However, previous research in our center demonstrated that a small part of CLTI-patients suffers from severe cognitive impairment [14,33] and they are mostly treated conservatively [59]. Although this assumption is derived from earlier research conducted in our own center and may subsequently limit generalizability, we believe the risk of selection bias due to this exclusion criterium will be low. Thirdly, in this study only CLTI-patients who are initially planned for revascularization are included. The possibility may occur, the degree of frailty of patients is initially underestimated by the vascular surgeon or during the general health screening. Patients in the prehabilitation group are assessed extensively during their visit to the MMPP, which may result in a higher level of frailty and a bigger risk of postoperative complications then initially presumed. Consequently, the vascular surgeon might suggest conservative treatment for these specific patients, according to the principle of ‘do no further harm’. However, this possibly leads to the risk of attrition bias in the prehabilitation group. Lastly, the two-week minimum duration of the prehabilitation program can be considered as a pragmatic necessity to ensure the targeted sample size can be reached. Previous observational research demonstrated the safety and benefits of a prehabilitation program for older CLTI-patients with a median duration of 23 days [33]. However, the overall treatment rationale of CLTI remains revascularization as soon as possible to ensure limb salvation in the Dutch vascular surgery practice. Implementing prehabilitation for older CLTI patients requires a substantial change in mindset and standard clinical practice.

In conclusion, this study will offer new insights on the effectiveness of a multimodal multicomponent prehabilitation program on length of stay, clinical and patient-reported outcomes, cost-effectiveness and the process of shared decision making in older CLTI-patients planned for revascularization.

## Supporting information

S1 File SPIRIT checklist POCI-study. (DOCX)

S2 File Study protocol POCI-study. (PDF)

S3 File Process evaluation questionnaires POCI-study. (DOCX)

S4 File Information brochure outpatient clinic. (PDF)
