# Supplementary material for: The effect of a multimodal multicomponent Prehabilitation program in Older adults with Chronic limb-threatening Ischemia (POCI-study): A study protocol for a multicenter randomized controlled trial
Source: PLoS One. 2026 Jul 29;21(7):e0354344. doi: 10.1371/journal.pone.0354344 (PMC13419218; doi:10.1371/journal.pone.0354344)
Supplement: S2 File — (PDF) [file pone.0354344.s002.pdf]

Nederlandse samenvatting protocol POCI-studie

Effect of multimodal ***P****rehabilitation* ***O****n* ***C****hronic limb-threatening* ***I****schemia (POCI-study)*

De **POCI-studie** is een studie naar het effect van prehabilitatie bij de oudere patiënt (≥ 65 jaar) met chronische ledemaat bedreigende ischemie (CLBI).

CLBI is de meest ernstige vorm van perifeer arterieel vaatlijden (PAV), die vooral op hogere leeftijd voorkomt en de kwaliteit van leven (KvL) ernstig vermindert. Ondanks steeds duurdere minimaal invasieve technieken (endovasculaire technieken) en operaties, en de daarmee gepaard gaande complicaties, is de kans op mortaliteit, afname van KvL en van het dagelijks functioneren hoog. Deze studie beoogt middels prehabilitatie, voorafgaand aan een interventie voor CLBI, een verbetering te geven in de (klinische) uitkomsten voor de patiënt, met name de duur van de ziekenhuisopname en de KvL. Verder worden een afname in zorgkosten en toename van verplaatste zorg naar huis beoogd. In dit project is er specifiek aandacht voor KvL van patiënt en mantelzorger, shared decision making (SDM) en de mantelzorgerbelasting. De ervaringen van de patiënt en de mantelzorger worden gebruikt bij de implementatie van de prehabilitatie. De studie opzet is multicenter en gerandomiseerd, met een kosteneffectiviteit analyse.

Patiënten boven de 65 jaar met CLBI die hiervoor een behandeling zullen ondergaan, worden bij presentatie in het ziekenhuis gerandomiseerd. Afhankelijk hiervan zullen zij wel of geen prehabilitatie krijgen, voorafgaand aan hun ingreep. Het prehabilitatie programma is een multidisciplinair programma en een samenwerking tussen de vaatchirurgie, geriatrie, fysiotherapie en diëtiek. Het prehabilitatie programma zal direct starten na randomisatie en duurt tenminste 2 weken, alvorens de interventie zal plaatsvinden. Eerder observationeel onderzoek in het Amphia ziekenhuis heeft aangetoond dat deze duur van prehabilitatie bij patienten met kritieke ischemie veilig kan worden toegepast. Middels uitgebreide screening en daarop aansluitend het aanbieden van (spier- en kracht) oefeningen, dieetadviezen, delierpreventieve maatregelen, suppletie van vitaminen en het behandelen van een preoperatieve anemie (indien van toepassing), wordt de patiënt geoptimaliseerd om in betere conditie de behandeling te ondergaan.

De studieopzet betreft evaluatie van een relatief nieuwe vorm van standaard zorg welke in het Amphia ziekenhuis reeds geïmplementeerd is, maar ook zal worden geïmplementeerd in het Elisabeth Tweesteden ziekenhuis en het Meander medisch centrum. De primaire uitkomstmaat is de lengte van de ziekenhuisopname. Secundaire uitkomstmaten zijn het aantal postoperatieve complicaties, de kwaliteit van leven van zowel patiënten als hun mantelzorgers en de lasten die mantelzorgers ervaren van de prehabilitatie. Tevens zal er een kosteneffectiviteitsanalyse worden verricht. Ten slotte zal er ook specifieke aandacht zijn voor de ervaringen en voorkeuren van patiënten en mantelzorgers ten aanzien van SDM.

RESEARCH PROTOCOL

POCI-study

Effect of multimodal prehabilitation on chronic limb-threatening ischemia

(OCTOBER 2025)

PROTOCOL TITLE Effect of multimodal prehabilitation on chronic limb-threatening ischemia

**Protocol ID**	NA
**Short title**	POCI-study
**Version**	1.3
**Date**	20-10-2025
**Coordinating investigator/project leader**	*Prof. Dr. L. van der Laan*<br>*Amphia Hospital, TIAS/TiU*<br>*Molengracht 21, Breda*<br>*lvanderlaan@amphia.nl*<br>*T 076 5954033*
**Principal investigator(s) (in Dutch: hoofdonderzoeker/ uitvoerder)**	*Amphia Hospital Breda:*<br>Prof. dr. L. van der Laan (PI)<br>Dr. M.C. Faes<br>Drs. S. Verbaan<br>Dr. E.T.A.M. van Delft<br><br>Prof. dr. P.W.H.E. Vriens, Elisabeth Tweesteden hospital, TiU<br>Dr. H. Jongsma, Meander Medical center<br>Prof. E.W. Steyerberg, Julius center UMC Utrecht<br>Dr. W.B. van den Hout, Leiden University
**Sponsor (in Dutch: verrichter/opdrachtgever)**	*Amphia Hospital*<br>*Molengracht 21, Breda*
**Subsidising party**	ZonMw – MedZo, Subsidieronde lijn 1: Passende en doelmatige zorg (Dossiernummer 11320102410007)
**Independent expert(s)**	*Dr. J.H. Smalberg*

	Amphia Hospital<br><br>*Molengracht 21, Breda*
**Laboratory sites**	*NA*
**Pharmacy**	*NA*

PROTOCOL SIGNATURE SHEET

Name	Signature	Date
**Sponsor or legal representative:**<br>*Dr. R. Wagenmakers*<br>*Amphia Hospital*<br>*Molengracht 21, Breda*		20-10-2025
**Coordinating Investigator/Project leader/Principal Investigator:**<br>*Prof. Dr. L. van der Laan, member of the department of Surgery*<br>*Amphia Hospital*<br>*Molengracht 21, Breda*<br>*Lvanderlaan@amphia.nl*<br>*T 076 5954033*		20-10-2025

TABLE OF CONTENTS

1.INTRODUCTION AND RATIONALE....11
2.OBJECTIVES....12
3.STUDY DESIGN....13
4.STUDY POPULATION....16
4.1Population (base)....16
4.2Inclusion criteria....16
4.3Exclusion criteria....16
4.4Sample size calculation....16
5.TREATMENT OF RESEARCH PARTCIPANTS....18
5.1Investigational product/treatment....18
5.2Use of co-intervention (if applicable)....18
5.3Escape medication (if applicable)....18
6.INVESTIGATIONAL PRODUCT....19
6.1Name and description of investigational product(s)....19
6.2Summary of findings from non-clinical studies....19
6.3Summary of findings from clinical studies....19
6.4Summary of known and potential risks and benefits....19
6.5Description and justification of route of administration and dosage....19
6.6Dosages, dosage modifications and method of administration....19
7.NON-INVESTIGATIONAL PRODUCT....20
7.1Name and description of non-investigational product(s)....20
7.2Summary of findings from non-clinical studies....20
7.3Summary of findings from clinical studies....20
7.4Summary of known and potential risks and benefits....20
7.5Description and justification of route of administration and dosage....20
7.6Dosages, dosage modifications and method of administration....20
7.7Preparation and labelling of Non Investigational Medicinal Product....20
7.8Drug accountability....20
8.METHODS....21
8.1Study parameters/endpoints....21
8.1.1Main study parameter/endpoint....21
8.1.2Secondary study parameters/endpoints....21
8.1.3Other study parameters....21
8.2Randomisation, blinding and treatment allocation....21
8.3Study procedures....21
8.4Withdrawal of individual research participants....25
8.4.1Specific criteria for withdrawal (if applicable)....26
8.5Replacement of individual research participants after withdrawal....26
8.6Follow-up of research participants withdrawn from treatment....26
8.7Premature termination of the study....26
9.SAFETY REPORTING....27
9.1Temporary halt for reasons of research participant safety....27

9.2AEs, SAEs 27
9.2.1Adverse events (AEs) 27
9.2.2Serious adverse events (SAEs) 27
9.3Follow-up of adverse events 28
9.4Data Safety Monitoring Board (DSMB) / Safety Committee 28
10.STATISTICAL ANALYSIS 29
10.1Primary study parameter(s) 29
10.2Secondary study parameter(s) 29
10.3Other study parameters 29
10.4Interim analysis (if applicable) 29
11.ETHICAL CONSIDERATIONS 30
11.1Regulation statement 30
11.2Recruitment and consent 30
11.3Objection by minors or incapacitated research participants (if applicable) 30
11.4Benefits and risks assessment, group relatedness 30
11.5Compensation for injury 30
12.The prehabilitation program under investigation in this study has already proven to be safe in practice (and has become a part of the standard care at the Amphia Hospital). Therefore, we kindly request exemption from the insurance obligation of the WMO (article 7, WMO). We are of the opinion that the study does not pose any additional risks to the participants, which is also supported by previous observational research. Of course, each participating centre does have a liability insurance. 30
12.1Incentives (if applicable) 30
13.ADMINISTRATIVE ASPECTS, MONITORING AND PUBLICATION 31
13.1Handling and storage of data and documents 31
13.2Monitoring and Quality Assurance 31
13.3Amendments 31
13.4Annual progress report 31
13.5Temporary halt and (prematurely) end of study report 31
13.6Public disclosure and publication policy 31
14.STRUCTURED RISK ANALYSIS 33
14.1Potential issues of concern 33
14.2Synthesis 33
15.REFERENCES 34

LIST OF ABBREVIATIONS AND RELEVANT DEFINITIONS

AE	Adverse Event
AFS	Amputation Free Survival
AR	Adverse reaction
ASA	American Society of Anesthesiologists
BMI	Body Mass Index
CCI	Charlson Comorbidity Index score
CCMO	Central Committee on Research Involving Human Subjects; in Dutch: Centrale Commissie Mensgebonden Onderzoek
CFS	Clinical Frailty Score
CGA	Comprehensive Geriatric Assessment
CLTI	Chronic Limb Threatening Ischemia
CSI	Caregiver Strain Index
DEMMI	The ‘De Morton Mobility Index’
DM	Decision Making
DOSS	Delirium Observation Screening Score
DSMB	Data Safety Monitoring Board
EPD	Electronic Patient Dossier
EQ-5D-5L	EuroQol 5 dimensions 5 level
GCP	Good Clinical Practice
GDPR	General Data Protection Regulation; in Dutch: Algemene Verordening Gegevensbescherming (AVG)
G8-score	Geriatric-8 score
IB	Investigator’s Brochure
IC	Informal Caregiver
IMDD	Investigational Medical Device Dossier
iMCQ	iMTA Medical Consumption Questionnaire
LOS	Length of Hospital Stay
METC	Medical research ethics committee (MREC); in Dutch: medisch-ethische toetsingscommissie (METC)
MIP	Maximal inspiratory and pressure
MMSE	The Mini-Mental State Examination
MNA-SF	Mini Nutritional Assessment – Short Form
MVE	Multidisciplinary Vascular surgical outpatient clinic for the Elderly
OS	Overall Survival

**PAD**	**Peripheral Arterial Disease**
**PICS**	**Perceived Involvement in Care Scale**
**PIF**	**Patient Information Folder**
**PTA**	**Percutaneous transluminal angioplasty**
**PTFE**	**Polytetrafluoroethylene**
**PVR**	**Pulse Volume Recording**
**QoL**	**Quality of Life**
**RCT**	**Randomized Controlled Trial**
**Review committee**	**Medical research ethics committee (MREC) or CCMO**
**(S)AE**	**(Serious) Adverse Event**
**SDM**	**Shared Decision Making**
**SNAQ-RC**	**Short Nutritional Assessment Questionnaire for the Residential Care**
**Sponsor**	**The sponsor is the party that commissions the organisation or performance of the research, for example a pharmaceutical company, academic hospital, scientific organisation or investigator. A party that provides funding for a study but does not commission it is not regarded as the sponsor, but referred to as a subsidising party.**
**SUSAR**	**Suspected Unexpected Serious Adverse Reaction**
**TASC-II**	**Trans-Atlantic Inter-Society Consensus Document on Management of Peripheral Arterial Disease II**
**TU**	**Tilburg University**
**TUG**	**Timed Up and Go**
**UAVG**	**Dutch Act on Implementation of the General Data Protection Regulation; in Dutch: Uitvoeringswet AVG**
**VNP**	**Vascular Nurse Practitioner**
**V-POSSUM**	**Vascular-Physiological and Operative Severity Score for the enUmeration of Mortality and Morbidity**
**WHOQoL-BREF**	**World Health Organization Quality of Life Brief Version**
**WMO**	**Medical Research Involving Human Subjects Act; in Dutch: Wet Medisch-wetenschappelijk Onderzoek met Mensen**

SUMMARY

Rationale: Chronic limb-threatening ischemia (CLTI) is the most severe form of peripheral arterial disease (PAD). CLTI primarily occurs in older adults and is associated with the need for (multiple) hospital admissions and significantly reduces the quality of life (QoL). Despite the increase of (more expensive) minimally invasive techniques (endovascular procedures) and surgeries, the associated complications, the risk of mortality, reduction in QoL, and decline in daily functioning remain high.

Objective: The aim of this study is to minimize the length of hospital stay (LOS) and improve patients' (clinical) outcomes, particularly their QoL, through prehabilitation prior to a CLTI intervention. Additionally, we aim to reduce healthcare costs and to improve the process of shared decision making (SDM).

Study design: This is a randomized multicentre study, with a cost-effectiveness analysis.

Study population: All CLTI patients aged 65 years or older presenting to the vascular surgeon with a new onset of CLTI who are planned for surgical or endovascular revascularization are screened for eligibility. Their primary IC will also be asked to participate in the study.

Intervention (if applicable): All patients undergo a general health screening by a vascular nurse practitioner (VNP) or researcher at the Multidisciplinary Vascular surgical outpatient clinic for the Elderly (MVE). Afterwards patients randomized to the intervention group are offered a prehabilitation program, starting directly at the MVE. Patients are referred to a physiotherapist and if indicated also to a geriatrician, dietician and a quit smoking coach. All will collaborate to optimize the patient's preoperative condition, to shorten the LOS and improve QoL of both patient and IC. In summary the prehabilitation program consists of: physical training, lifestyle advice, diet advice and supplements, iron infusion (Ferinject) in case of an iron deficiency anaemia and a comprehensive geriatric assessment (CGA).

Main study parameters/endpoints: The primary outcome is the LOS. Secondary outcomes will be QoL of the patient and their IC, 6-month mortality, postoperative complications, hospital readmission, 2level of perceived SDM and preferred participation in SDM of the patient and their IC, perceived and preferred roles in decision making (DM) of the patient and their IC and cost-effectiveness.

Nature and extent of the burden and risks associated with participation, benefit and group relatedness: The burden for all participants consists of the visit to the MVE, drawing a blood sample and filling in questionnaires at baseline, at the second visit to the vascular surgeon and 6 months after revascularization. Patients in the intervention group are provided with home-based exercises by a physiotherapist. These exercises take time and should be performed daily and written down in a diary. If indicated they also receive dietary advice and advices from the geriatrician to take into account at home.

1. INTRODUCTION AND RATIONALE

In the face of an aging population, the prevalence of CLTI among elderly persons of 65 years and older has become a major public health concern. CLTI, a severe form of PAD, is characterized by atherosclerotic occlusion of the arteries supplying the lower extremities, leading to ischemic rest pain and/or tissue damage (1). Annually, 500-1000 new cases of CLTI occur per 1 million people in Europe.

Revascularization is the primary treatment for CLTI and current guidelines advice to do this as soon as possible to prevent major limb amputation. However, the treatment of CLTI is associated with reduced QoL of both patient and their ICs, long duration of hospital stays, intensive wound care, high chance of discharge to a nursing home and places a high burden on the IC (2). Thereby, up to a quarter of the CLTI patients is at risk for major amputation despite revascularization attempts.

Recognizing the need for a strategy to minimize the risk of developing complications, diminish the LOS and increase QoL, multimodal prehabilitation has emerged as a promising preoperative approach within vascular surgery. Prehabilitation is the all-encompassing term of preoperative optimization of the patient’s functional capacity and resilience, in order to reduce postoperative complications (3). Interventions include physical, mental, functional and nutritional optimization.
The decision on the treatment of CLTI should be made in a SDM process. SDM increases patients’ knowledge of treatment options, initiates accurate risk perception, improves patient satisfaction and reduces undesired care in certain categories of patients (4). However, it is known that SDM is still not common practice in vascular surgery and thorough research in this field is lacking.

Our research group has extensive experience with prehabilitation, and demonstrated that prehabilitation in older CLTI patients is safe (5-7). This previous research informed a paradigm shift in vascular surgery, from revascularization as soon as possible, to time for prehabilitation. However, the research was monocenter and observational. A multicenter randomized controlled trial (RCT) and cost-effectiveness study is lacking. Therefore, this POCI-study is designed, a multicenter RCT studying the effect of prehabilitation on LOS, postoperative complications, hospital readmission and QoL, including a cost effectiveness analysis. In addition, we aim to investigate the experiences and preferences of both patients and ICs with (S)DM concerning CLTI treatment in the outpatient vascular surgical clinic.

2. OBJECTIVES

Primary Objective:
The aim of this study is to minimize the LOS in elderly patients of 65 years and older with CLTI that require surgical or endovascular revascularization. Our hypothesis is that by implementing a prehabilitation program focused on preoperative optimization of the patient's functional capacity, the LOS will decrease with 25% compared to patients not attending the program.

Secondary Objective(s):
Secondary, we aim to reduce the number of postoperative complications, the amount of hospital readmissions,6-month mortality and the burden on the IC (using the Caregiver Strain Index (CSI)). In addition, our goal is to improve the QoL of both patient and their IC (QoL, measured by using the WHOQoL-BREF and the health related QoL measured by using the EQ-5D-5L). The level of perceived SDM of the patient and their IC (using the CollaboRATE), perceived and preferred roles in decision making (DM) of the patient and their IC (using the Adapted Control Preference Scale (ACPS)) and preferred participation in SDM of the patient and their IC (using the Perceived Involvement in Care Scale (PICS)) will be measured. Lastly, we aim to reduce health-care costs by implementing the prehabilitation program.

3. STUDY DESIGN

An RCT with a cost-effectiveness analysis, will be performed in three non-university large teaching hospitals in the Netherlands (Amphia Hospital Breda, Elisabeth Tweesteden Hospital Tilburg and Meander Medical Center Amersfoort), during the period of November 2025 to May 2028. All patients of 65 years or older, with a new onset of CLTI presented at the outpatient clinic of the vascular surgeon, who are planned for surgical or endovascular revascularization and who meet the in- and exclusion criteria are asked to participate in the study. In addition, their primary IC will also be requested to take part in the study. A primary IC is defined as follows: the person most involved in caring for the CLTI patient and supporting at least one instrumental activity of daily living of the patient or defined as the contact who supports in decisions by the patient. Patients can still be included, if their primary IC refuses participation. However, primary ICs can only be included together with a patient.
At the outpatient clinic information about the POCI-study is provided by a VNP or researcher and patients and ICs receive an information folder. They are called by telephone the next day by a researcher orVNP to ask if they want to participate. If so, oral informed consent will be obtained by the researcher or VNP and patients are randomized immediately upon inclusion, either into the prehabilitation group or the control group, the latter receiving the standard of care. Their IC will automatically be randomized to the same group. Within a few days all patients undergo a general health screening by a VNP or researcher and a blood sample will be taken as part of the standard care. Written informed consent will be obtained during this visit. Afterwards patients receive three questionnaires: the WHOQoL-BREF, the EQ-5D-5L and the iMTA Medical Consumption Questionnaire (iMCQ) (t=0). ICs are also asked to fill in two questionnaires: the WHOQoL-BREF, and the CSI (t=0). Afterwards, patients randomized into the intervention group are referred to the prehabilitation program where they are assessed by a physiotherapist and if indicated also a geriatrician, dietician or a quit smoking coach. The prehabilitation program will take two weeks, considering both the time needed to possibly reach significant results with the program and the time in which treatment is planned and must take place.

After treatment planning is complete, the vascular surgeon will discuss this plan with patients and their IC in a second visit to the outpatient clinic. Afterwards both patient and IC are asked to fill in three questionnaires, regarding the SDM process: the CollaboRATE, the ACPS and the PICS (tSDM=0).

Patients and their ICs will be included during a period of 2 years and will stay in follow-up for 6 months. At the 6-months follow-up patients are asked to fill in four questionnaires: the

WHOQoL-BREF, the EQ-5D-5L, the iMCQ and a process evaluation form designed by the researchers (t=1). ICs are asked to fill in three questionnaires: the WHOQoL-BREF, the CSI and a process evaluation form (t=1).

Inclusion of the patient and their IC will be on the day after the first outpatient clinic visit. This way the prehabilitation starts within a few days, parallel to the treatment planning (imaging, multidisciplinary meeting, planning for surgery or endovascular treatment) and there will be no delay in the treatment schedule.

FIGURE 1 – FLOWCHART POCI-STUDY

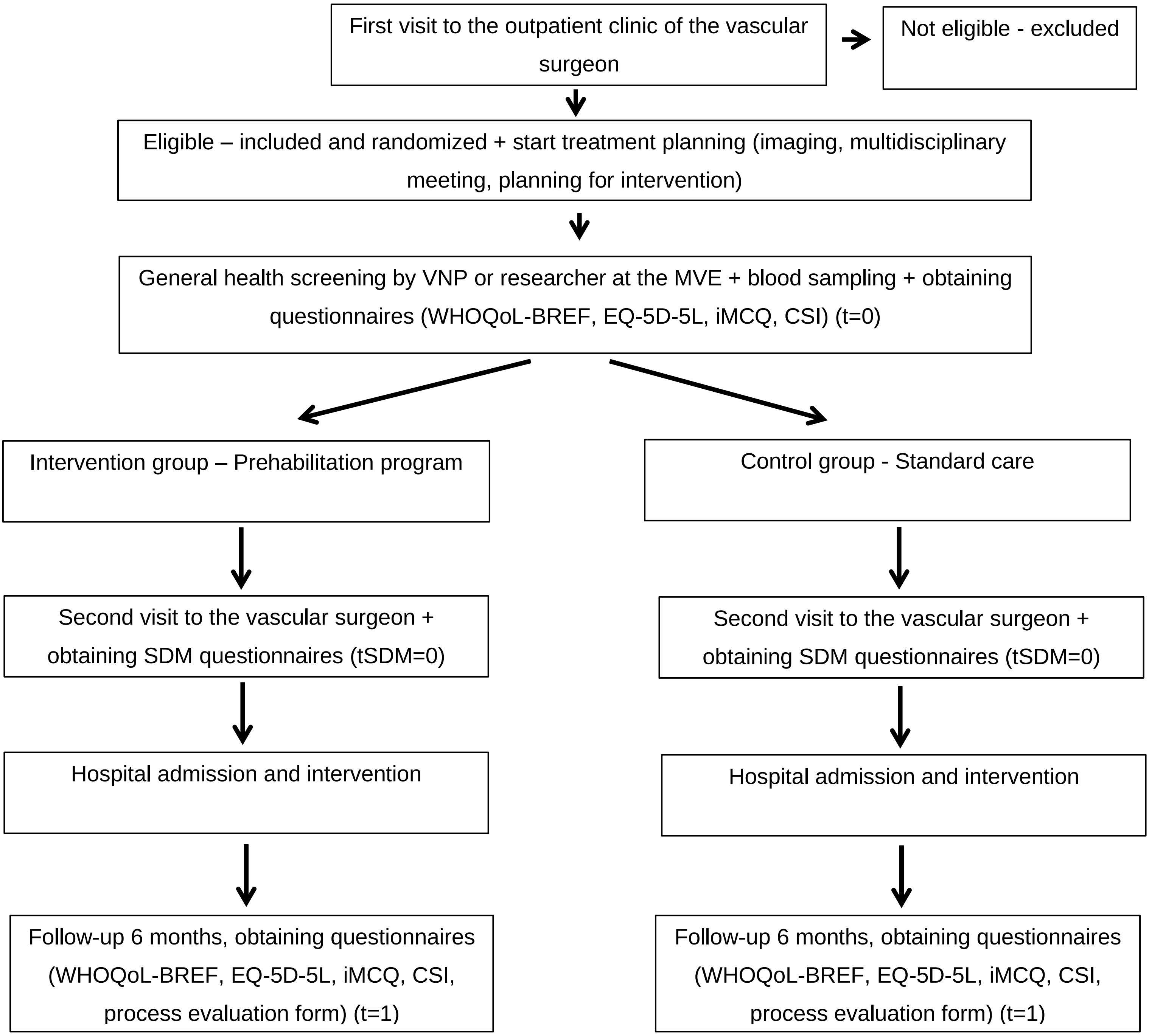

4. STUDY POPULATION

4.1 Population (base)

All patients in the Amphia Hospital (Breda), the Elisabeth Tweesteden Hospital (Tilburg) and the Meander Medical center (Amersfoort), referred by a general practitioner with suspected CLTI, will be seen on the outpatient clinic by a vascular surgeon. Diagnosis of CLTI is based on impaired perfusion combined with a Rutherford classification of either stage 4 or 5 (8). The severity of the CLTI is scored by the vascular surgeon by using the WIfI score (9). All CLTI patients exceeding the age of 65 with a WIfI-score of Wounds (W) ≤2 and foot infections (fI) ≤2 are screened for eligibility according to the in- and exclusion criteria stated below. Additional imaging is requested. If eligible, patients are offered the study information and asked to participate in this study. Their primary IC will be asked to participate with the patient.

4.2 Inclusion criteria

In order to be eligible to participate as a patient in this study, a participant must meet all of the following criteria:

- Aged 65 years or older
- Diagnosis of CLTI (Chronic Limb Threatening Ischemia), based on:
    - Anamnestic complaints of ischemic rest pain or night pain (Rutherford stage 4) OR (Minor) tissue loss, non-healing ulcer and/or focal gangrene with diffuse pedal ischemia (Rutherford stage 5) (8)
      AND

Impaired perfusion, quantified by a flat or barely pulsatile ankle or metatarsal pulse volume recording (PVR), a low resting ankle pressure, a low ankle brachial index and/or a low toe pressure.

In order to be eligible to participate as an IC in this study, a participant must meet the following criterium:

- IC of a CLTI patient of 65 years or older

4.3 Exclusion criteria

A potential participant who meets any of the following criteria will be excluded from participation in this study:

- Patients undergoing conservative therapy
- Need for urgent surgery or endovascular therapy (<2 weeks), based on either:
    - WIfI-score of W = 3
    - WIfI-score of fI = 3
- Patients or ICs who are unable to complete questionnaires, due to either lingual or cognitive incompetence.

4.4 Sample size calculation

In this multicenter study we aim for a total of 300 patients, 100 patients from each hospital. With this sample size we have 80% statistical power to test hypotheses with 2-sided alpha set at 5% for the following differences between randomized groups.

Length of hospital stay: The calculation of the effect size is based on the difference in means with SD. In a previous study (7), prehabilitation was associated with a difference of 2 vs over 4 days on average. A halving in length of stay requires 54 patients in total (27 per group, assuming a SD of log (LOS) of 0.9, as in the previous study). With 300 patients, a decrease by a factor 0.75 (so 25% reduction in LOS) provides 80% power.

For complications, we will analyze minor and major complications. In the previous study (7), prehabilitation was associated with the following reductions in complications: minor, 26 vs 14%; major 23 vs 18%. These differences would require sample sizes of 346 and over 2000 patients in total respectively. With 300 patients we will have reasonable power for detecting a difference in minor complications (power 69%), but insufficient statistical power for a difference of 23 vs 18% in major complications (power 15%). These calculations were done using the online sample size calculators at https://sample-size.net/

5. TREATMENT OF RESEARCH PARTCIPANTS

In this study we will provide a multidisciplinary prehabilitation program to patients that are planned to undergo endovascular or surgical revascularization for CLTI. The duration of the prehabilitation program is at least 2 weeks and consists of seven major components:

1) Screening of general health, signs of frailty, comorbidities, nutritional state, and performing functional and cognitive testing, by a vascular nurse practitioner or researcher at the MVE.

All patients included in the study, regardless of control or intervention group, will go through this first step of the MVE. Afterwards the patients in the intervention group start their prehabilitation program, consisting of:

2) Screening and provision of personalized, home-based exercises by a physiotherapist. Patients will track their daily activities in a diary.

And, if indicated:

3) Provision of nutritional advice by a dietician, based on the body mass index (BMI), Mini Nutritional Assessment Short Form (MNA-SF) score and the SNAQ-score. If indicated, patients will receive high protein nutrient drinks and recommendations of a high protein diet.

4) Comprehensive geriatric assessment (CGA) by a geriatrician.

5) In case of an iron deficiency anemia (haemoglobin <8,1 mmol/L for male patients and <7,4 mmol/L for female patients), patients will receive iron infusion using ferric carboxymaltose,

6) Assessment of self-reliance and home situation by a prearranged homecare nurse

7) Guidance and support for smoking cessation by a quit smoking coach.

The IC participates with the patient in the program. We performed a pilot interview study in CLTI patients and their ICs. Their advises and recommendations are incorporated in our prehabilitation program for CLTI and the POCI-study in general.

5.1 Investigational product/treatment

Not applicable

5.2 Use of co-intervention (if applicable)

There are no restrictions for participants.

5.3 Escape medication (if applicable)

Not applicable

6. INVESTIGATIONAL PRODUCT

6.1 Name and description of investigational product(s)

Not applicable.

6.2 Summary of findings from non-clinical studies

Not applicable.

6.3 Summary of findings from clinical studies

Not applicable.

6.4 Summary of known and potential risks and benefits

Not applicable.

6.5 Description and justification of route of administration and dosage

Not applicable.

6.6 Dosages, dosage modifications and method of administration

Not applicable.

7. NON-INVESTIGATIONAL PRODUCT

7.1 Name and description of non-investigational product(s)
Not applicable.

7.2 Summary of findings from non-clinical studies
Not applicable.

7.3 Summary of findings from clinical studies
Not applicable.

7.4 Summary of known and potential risks and benefits
Not applicable.

7.5 Description and justification of route of administration and dosage
Not applicable.

7.6 Dosages, dosage modifications and method of administration
Not applicable.

7.7 Preparation and labelling of Non-Investigational Medicinal Product
Not applicable.

7.8 Drug accountability
Not applicable.

8. METHODS

8.1 Study parameters/endpoints

8.1.1 Main study parameter/endpoint

The primary outcome of this study is LOS (Appendix 1-Uitkomstmaten).

8.1.2 Secondary study parameters/endpoints

Secondary outcomes (Appendix 1-Uitkomstmaten) will be postoperative complications, hospital readmission rate, 6-month mortality, QoL of both patient and their informal caregiver (measured by using the WHOQoL-BREF), the health related QoL of patients (measured by using the EQ-5D-5L), burden on informal caregiver (using the CSI), the level of perceived SDM of the patient and their IC (using the CollaboRATE), perceived and preferred roles in DM of the patient and their IC (using the ACPS), preferred participation in SDM of the patient and their IC (using the PICS), a cost-effectiveness analysis will be performed (using the iMCQ) and a process evaluation will take place (using an effect evaluation form designed by the research group). For the forms used to measure these endpoints, see ‘F1-Vragenlijsten.

8.1.3 Other study parameters

Not applicable

8.2 Randomisation, blinding and treatment allocation

Randomization will be performed immediately after inclusion of a participant, either by the VNP or by the responsible researcher. Participants will be allocated to either the prehabilitation group or the control group. A variable block randomization procedure will be applied to minimize the risk of unequal group sizes and to reduce predictability of assignments. To further ensure comparability between study arms, randomization will be stratified by the participating hospital (to eliminate possible differences between hospitals). By stratifying we aim to prevent systematic imbalances and to enhance the internal validity of the trial. The randomization procedure will be carried out using the validated electronic data capture system Castor EDC. This study is not subject to blinding due to the extensive and active nature of the intervention. While blinding is not feasible, objective outcome measures and standardized assessment procedures will be used to minimize potential bias.

8.3 Study procedures

In this study we will provide a multidisciplinary prehabilitation program to patients ≥ 65 years that are planned to undergo endovascular or surgical revascularization for CLTI. The prehabilitation program is started at the MVE and will have a total duration of +/- 2 weeks. This cut-off point is chosen taking in account both the time that we expect to get most optimal results from the program, and the time wherein revascularization should take place.

<u>First visit to the outpatient clinic of the vascular surgeon (diagnosis)</u>

All patients will be examined by the vascular surgeon at the first outpatient clinic visit. The WIfI score will be used to classify the patient at the time of initial presentation (see Appendix 2-Meetinstrumenten). This score for threatened lower limbs combines the three main factors that have an impact on limb amputation risk: Wound (W), Infection (I) and foot Infection (fI) (9). This score is helpful in estimating the benefit of revascularization and the risk of amputation at one year for each individual patient.

Additional research will be requested. This may include an Electrocardiogram (ECG), Ankle-Brachial Index (ABI), toe pressure in case of non-compressible vessels (common in diabetics). Imaging may be performed by duplex, or Magnetic Resonance Angiography (MRA) or Computed Tomography Angiogram (CTA). Vascular pathology will be scored using the Trans-Atlantic Inter-Society Consensus Document on Management of Peripheral Arterial Disease II (TASC-II, see Appendix 2-Meetinstrumenten) and description of the crural outflow (in 1,2 or 3 arteries) (10-12).

All patients meeting the inclusion criteria for this study and their IC receive an information map, consisting of an (patient) information folder (PIF) and an Informed consent form and are called the next day by a researcher or VNP to ask if they want to participate in the study (E1.Proefpersonen informatiebrief and E1.Mantelzorger informatiebrief).

<u>Multidisciplinary Vascular surgical outpatient clinic for the Elderly (MVE)</u>

Directly after inclusion patients will be referred to the MVE. It is important that the patient brings an informal caregiver, for example a partner, family member or close friend to optimize the (hetero)anamnesis and to make sure all information is understood and processed well. Also, hereby we can investigate the possibility of informal care, provided by friends or family. If needed, home care will already be arranged for after discharge. Written informed consent for participating in the study will be obtained by a researcher or VNP.

- *Vascular surgery Nurse Practitioner or researcher*

A trained VNP or researcher will screen all patients on baseline characteristics, signs of frailty and the presence of risk factors for delirium. Comorbidity will be scored using the V-POSSUM score, ASA-classification, Charlson comorbidity index and the Comprehensive Comorbidity Index (CCI) (Appendix 2-Meetinstrumenten). Frailty will be scored using the Clinical Frailty Scale (CFS) (13) and the Geriatric-8-score (G8-score) (14). The VNP or researcher will withdraw the Mini Mental Status Exam (MMSE) (15) to assess cognitive function. Dietary state is assessed by using the Short Nutritional Assessment Questionnaire (SNAQ-score) (16). Also, an inventory is made of available (informal) care and whether problems are expected after dismissal. Patients randomized to the intervention group, might subsequently be referred to the geriatrician, dietician and a 'quit smoking coach' if necessary.

- *Biochemistry tests*

Biochemistry tests will be performed prior to the visit to the VNP or researcher at the MVE. This will consist of: haemoglobin, haematocrit, MCV, C-Reactive Protein, leukocytes, thrombocytes, liver enzymes (ASAT, ALAT), electrolytes (sodium, potassium, chloride), albumin, MDRD, urea, creatinine, INR, lipid spectrum including cholesterol, vitamin D, vitamin B12, folium acid, transferrin, ferritin. Therefore, three blood tubes will be taken.

- *Physiotherapist*

After the visit to the VNP or researcher, patients in the intervention group will be screened by a physiotherapist. The physical state of the patient will be examined and the following tests are performed:

- Maximal inspiratory and pressure (MIP);
- The 'De Morton Mobility Index' (DEMMI)
- Gripstrength, using the hand dynamometer (JAMAR®);
- Timed Up and Go (TUG) test;
- Timed Chair-Stand test;
- Gait speed (10 Meter walking test);

Based on these results, patients are provided with their own instructions and home-based exercises (Appendix 2-Meetinstrumenten). Patients are asked to keep track of all their activities in a daily diary.

- *Optional: Geriatrician*

If indicated, patients will undergo a CGA as performed by a geriatrician to assess frailty. Based on the CGA, the geriatrician will, among other things, define preventive measures for functional decline and delirium. Patients will be referred to the geriatrician in case of:

- MMSE< 26
- TUG > 14 sec
- Previous delirium
- Polypharmacy > 4 (cardiovascular risk management medications not included)
- Alcohol consumption > 7 units per week
- G8-score < 14

- *Optional: dietician*

If indicated, a dietician will evaluate the nutritional status of the patient (using the MNA-SF score and the SNAQ-score). If indicated, they will receive high protein nutrient drinks and recommendations of a high protein diet.

- *Optional; quit smoking coach*

If indicated, patients will be referred to a ‘quit smoking coach’ to receive guidance in reducing nicotine abuse. They will see the patients within 3 days after referral and offer appropriate guidance on an individual basis or group therapy.

- *Optional; iron infusion*

In case of iron deficiency anemia (haemoglobin <8,1 mmol/L for male patients and <7,4 mmol/L for female patients), patients will receive iron infusion using Ferinject (ferric carboxymaltose). This will clinically take place during a day-care admission. Patients will receive a dose of 1000mg intravenous in 15 minutes.

Multidisciplinary meeting

All patients will be discussed in a weekly, multidisciplinary meeting with vascular surgeons and (intervention)radiologists. Based on the type of pathology and taking in account both the imaging and the mental state, condition, frailty, V-POSSUM and comorbidity of the patient as screened during the MVE visit, consensus will be made concerning the type of treatment these patients will undergo. Regarding revascularization for CLTI there are several options:

Surgical options cover:

- endarterectomy (femoral communal artery) with or without percutaneous transluminal angioplasty (PTA) of any trajectory
- bypass
  - supragenual, infragenual, crural

- venous (using the great saphenous vein, small saphenous vein or arm vein) or polytetrafluoroethylene (PTFE)

- aorto-iliacal or femoral.
- may include wound debridement and minor amputation

Endovascular options cover:

- PTA of any trajectory
- with or without a drug eluting balloon
- with or without stenting
- may include wound debridement and minor amputation

Patients treated conservatively and patients requiring primary major amputations are not included in this study.

Second visit to the outpatient clinic (treatment)

After treatment planning is complete, the vascular surgeon will discuss this plan with patients and their IC in a second visit to the outpatient clinic. Afterwards both patient and IC are asked to fill in three questionnaires, regarding the SDM process: the CollaboRATE, the ACPS and the PICS (tSDM=0).

Clinical admission

At the time of admission, after +/- 2 weeks, a process analysis will be performed by using a process evaluation form designed by the researchers (F1-Vragenlijsten). We will test compliance and evaluate whether the given interventions and/or instructions were performed at home. Patients diaries are used as a guideline throughout this evaluation.

SDM Information Brochure

To evaluate the experiences and preferences regarding SDM of patients and their ICs all patients and their ICs are asked to fill in the CollaboRATE, ACPS and the PICS. Specific information brochures concerning the prehabilitation program for patients will be actualized (E3.Informatiemap Prehabilitatie Polikliniek). After three months and at the end of the project the information brochures will be evaluated with patients (if possible) and ICs. Furthermore, an existing patient education video on shared decision making (Divi, Indiveo) will be adjusted for making decisions in CLTI treatment. The video is intended for both patients and ICs. The adjustment of the video will be done in cooperation with patients and ICs.

Follow-up at 6 months

At 6 months after revascularization we will again ask patients and their ICs to fill in the WHOQOL-BREF. Patients are also asked to fill in the EQ-5D-5L questionnaire and the iMCQ. The IC is also asked to fill in the CSI. In addition, both patient and IC are asked to fill in an additional questionnaire regarding process evaluation.

8.4 Withdrawal of individual research participants

Participants can leave the study at any time for any reason without any consequences if they wish to do so. Already collected data will be included in the study. Data regarding LOS, mortality, hospital readmission rate and complications will still be collected from the electronic patient dossier (EPD). The investigator can decide to withdraw a participant from the study for urgent medical reasons.

8.4.1 Specific criteria for withdrawal (if applicable)

Patients will be excluded from the study if they refuse to participate in the prehabilitation program or fill in the questionnaires (with any reason).

Patients will be noted ‘lost to follow-up’ if they are excluded after revascularization (with any reason). They will not be asked to fill in the questionnaires at 6 months but follow the common follow-up trajectory. All saved content will be kept and used in the study.

8.5 Replacement of individual research participants after withdrawal

Withdrawn subjects will not be replaced.

8.6 Follow-up of research participants withdrawn from treatment

Subjects withdrawn from treatment will participate in the usual follow-up. LOS, complications, hospital readmission and mortality will be noted.

8.7 Premature termination of the study

This study will be terminated prematurely in case of any remarkable negative effects (complications and/or mortality) in the intervention group, compared to the control group.

9. SAFETY REPORTING

9.1 Temporary halt for reasons of research participant safety

In accordance to section 10, subsection 4, of the WMO, the sponsor will suspend the study if there is sufficient ground that continuation of the study will jeopardise participant health or safety. The sponsor will notify the review committee without undue delay of a temporary halt including the reason for such an action. The study will be suspended pending a further positive decision by the review committee. The investigator will take care that all participants are kept informed.

9.2 AEs, SAEs

We want to emphasize that the intervention of this study is already proven safe in CLTI patients (5-7). We don’t expect any AE, SAE or SUSARs to take place as a result of this program. The only possibility is an AE or SAE regarding the extra physical activity we ask patients to perform (e.g. when a patient falls and fractures a hip during an extra exercise).

9.2.1 Adverse events (AEs)

Adverse events are defined as any undesirable experience occurring to a participant during the study, whether or not considered related to the prehabilitation program. All adverse events reported spontaneously by the participant or observed by the investigator or his staff will be recorded.

9.2.2 Serious adverse events (SAEs)

A serious adverse event is any untoward medical occurrence or effect that

- results in death;
- is life threatening (at the time of the event);
- requires hospitalisation or prolongation of existing inpatients’ hospitalisation;
- results in persistent or significant disability or incapacity;
- is a congenital anomaly or birth defect; or
- any other important medical event that did not result in any of the outcomes listed above due to medical or surgical intervention but could have been based upon appropriate judgement by the investigator.

An elective hospital admission will not be considered as a serious adverse event.

All events will be reported by the investigator to the sponsor. Patients will still be included in our database and will not be withdrawn from this study in case an adverse event occurs. To our knowledge, no other adverse events might occur when participating in this study.

The sponsor will report the SAEs through the web portal ToetsingOnline to the accredited METC that approved the protocol, within 7 days of first knowledge for SAEs that result in death or are life threatening followed by a period of maximum of 8 days to complete the initial preliminary report. All other SAEs will be reported within a period of maximum 15 days after the sponsor has first knowledge of the serious adverse events.

9.3 Follow-up of adverse events

All AEs will be followed until they have abated, or until a stable situation has been reached. Depending on the event, follow up may require additional tests or medical procedures as indicated, and/or referral to the general physician or a medical specialist. SAEs need to be reported till end of study within the Netherlands, as defined in the protocol.

9.4 Data Safety Monitoring Board (DSMB) / Safety Committee

An independent DSMB will be established. The members of the DBMS will be:

- Prof. Dr. H.J.M. Verhagen, vascular surgeon, Erasmus Medical Center
- Dr. M. de Leeuw, epidemiologist, Amphis Hospital
- S. van den Bosch, scientific advisor/monitor, Amphia Hospital

None of the members are otherwise involved in the study and the members have no conflict of interest with the trial. The DSMB will assess the safety of the tested prehabilitation component during the trial. Interim analysis for the DSMB meetings shall be focused on complications. Data will be presented as a percentage.

Based on this data, the DSMB will recommend one of the following actions:

- No action needed, trial continues as planned
- Proposing protocol changes
- Early stopping due to safety concerns

The advice(s) of the DSMB will only be sent to the sponsor of the study. Should the sponsor decide not to fully implement the advice of the DSMB, the sponsor will send the advice to the review committee, including a note to substantiate why (part of) the advice of the DSMB will not be followed.

10. STATISTICAL ANALYSIS

Statistical analyses are performed using IBM SPSS statistical software (SPSS Inc., Chicago, Illinois, USA). A two-sided p-value of less than 0.05 will be considered statistically significant. Adjusted analysis with prognostic baseline characteristics (e.g. gender, age) will be performed using regression analysis. This analysis corrects for baseline differences, optimizes statistical power, and provides better individualized treatment effect estimates. Missing data will be studied for specific patterns of occurrence, and multiply imputed. A power analysis with sample size calculation is performed, as described in paragraph 4.4 (sample size calculation).

Results of categorical data variables are described in frequencies with percentages and differences will be tested using the Chi-square test. Continuous data are described as median (interquartile range) and Mann-Whitney U-tests will be performed to test for group differences. Ordinal variables will be tested using the Mann-Whitney U-test. Data analysis will be done according to the intention-to-treat concept, which implies that all patients are included in the analysis irrespective of whether they completed the prehabilitation program. Secondary, a per protocol analysis will be performed, although interpretability may be difficult for this analysis. All epidemiological issues and statistical methods will be discussed with Prof. Dr. E.W. Steyerberg, a member of the research group.

10.1 Primary study parameter(s)

Primary endpoint of the study is the LOS. LOS will be extracted from the electronic patient dossiers (EPD). We will use a linear regression model for important covariates (such as age, gender, surgical history, comorbidities, use of medication, smoking status, use of alcohol, home situation) that may contribute to the outcome of LOS. We expect the continuous variable LOS to be skewed, therefore we will perform a log-transformation. Hospital site will be a factor in the regression analysis. This way stratification will take place for each hospital site.

10.2 Secondary study parameter(s)

As described in chapter 8.1 secondary study parameters will be postoperative complications, hospital readmission rate, 6-month mortality, the QoL of both patient and their informal caregiver (measured by using the WHOQoL-BREF), health related QoL of patients (measured using the EQ-5D-5L), burden on IC (using the CSI), the level of perceived SDM of the patient and their IC (using the CollaboRATE), perceived and preferred roles in DM of the patient and their IC (using the ACPS) and preferred participation in SDM of the patient and their IC (using the PICS). Continuous outcomes are analyzed using a linear regression model, whereas binary outcomes are analyzed by using a logistic regression model. Correction for important covariates (such as age,

gender, surgical history, comorbidities, use of medication, smoking status, use of alcohol, home situation) will take place. Hospital site will be a factor in the regression analysis. Repeated measures will be analyzed with mixed effect models with random effects per patients to address within patient clustering.

Lastly a cost-effectiveness analysis will be performed. The economic evaluation will include a cost analysis from hospital perspective, a cost-utility analysis from societal perspective (CUA, i.e. costs per QALY), and a budget impact analysis (BIA). In all three analyses, care with and without prehabilitation will be compared according to intention to treat. For the hospital cost analysis, 6 months interventions and stay will be assessed from the financial records of the Amphia Hospital, Meander Medical centre and Elisabeth TweeSteden hospital. For the CUA, also costs outside the hospital will be included, such as wound care, physiotherapy, home care, and informal care. These will be assessed using shortened versions of the iMCQ questionnaire (filled out at 6 months, over the preceding six months). Care will be valued according to Dutch reference prices, including travel costs. Productivity costs will not be included, because of the low labor participation in this 65+ study population. QALYs will be calculated using the Dutch tariff for the five-level EuroQoL EQ-5D (assessed at 0, and 6 months), with a sensitivity analysis using the EuroQol visual analogue scale. Incremental average 6 months costs and QALYs will be compared using net-benefit analysis, with multiple imputation to account for missing data. The BIA will estimate the financial impact of different implementation scenarios at the national level. The analysis will be conducted from the perspectives of society, hospital and insurers, using the ZonMw BIA tool. The BIA will be based on the costs as estimated during the study, and the expected numbers of patients in the Netherlands. Healthcare will be valued according to cost prices (for the societal perspective) or NZa prices (for the hospital and insurer perspectives). Costs will be estimated per 1-year budget period for a time horizon of 5 years, assuming 50% to 100% implementation after 4 years.

10.3 Other study parameters

Not applicable.

10.4 Interim analysis (if applicable)

Interim analysis for the meetings of the DSMB shall be focused on the question if the complication ratio is not increased in the non prehabilitation group compared to the prehabilitation group.

11. ETHICAL CONSIDERATIONS

11.1 Regulation statement

The study will be conducted according to the principles of the Declaration of Helsinki (64th World Medical Association General Assembly, Brazil, October 2013) and in accordance with the Medical Research Involving Human Subjects Act (WMO). This study will be performed with approval by the medical research ethics committee (MREC); in Dutch: Medisch Ethische Toetsings Commissie (METC). Approval of the METC will be requested as soon as possible after submission of this project plan. Participation in this study will be on a voluntary basis. If patients do not wish to participate, they can do so without specifying why. Deciding not to participate in the study will not affect regular treatment and follow-up care. Participants will be allowed to withdraw from the study at any time after they have given their written consent.

11.2 Recruitment and consent

Patients and their IC will be asked to give written informed consent while visiting the MVE by the VNP or a researcher. The patient information letter, the informal caregiver information letter and the informed consent forms for patients and their IC are attached (E1.Proefpersonen informatiebrief and E.1Mantelzorger informatiebrief).

11.3 Objection by minors or incapacitated research participants (if applicable)

Not applicable

11.4 Benefits and risks assessment, group relatedness

The only burden of this study may be the time it takes patients and their IC to visit the MVE, fill in the questionnaires, which we kept brief in order to keep the burden as small as possible and the time it takes to do the home-based exercises. We expect no to minimal risk for adverse events.

11.5 Compensation for injury

The prehabilitation program under investigation in this study has already proven to be safe in practice (and has become a part of the standard care at the Amphia Hospital). We are of the opinion that the study does not pose any additional risks to the participants, which is also supported by previous observational research. However, each participating centre has a liability insurance. Additionally, Amphia has a participant’s insurance.

11.6 Incentives (if applicable)

Not applicable

12. ADMINISTRATIVE ASPECTS, MONITORING AND PUBLICATION

12.1 Handling and storage of data and documents

Data will be handled confidentially and collected from the electronic patient dossier (EPD). A specific study folder will be created in which data is saved. Questionnaires are securely safeguarded by the researcher. Organization and storage of data and questionnaires will be done using Castor SMS and Castor EDC, a clinical data management platform. The handling of personal data will be in line with the Dutch Act on Implementation of the General Data Protection Regulation (in Dutch: Uitvoeringswet AVG, UAVG).

12.2 Monitoring and Quality Assurance

Monitoring of informed consent and data will take place by the project leader, principal investigators and PhD student.

12.3 Amendments

Amendments are changes made to the research after a favourable opinion by the review committee has been given. All amendments will be notified to the review committee that gave a favourable opinion.

Non-substantial amendments will not be notified to the review committee, but will be recorded and filed by the sponsor.

12.4 Annual progress report

The sponsor/investigator will submit a summary of the progress of the trial to the review committee once a year. Information will be provided on the date of inclusion of the first participant, numbers of participants included and numbers of participants that have completed the trial, serious adverse events, other problems, and amendments.

12.5 Temporary halt and (prematurely) end of study report

The investigator/sponsor will notify the review committee of the end of the study within a period of 8 weeks. The end of the study is defined as the last patient's last visit.

The sponsor will notify the review committee immediately of a temporary halt of the study, including the reason of such an action.

In case the study is ended prematurely, the sponsor will notify the review committee within 15 days, including the reasons for the premature termination.

Within one year after the end of the study, the investigator/sponsor will submit a final study report with the results of the study, including any publications/abstracts of the study, to the review committee.

12.6 Public disclosure and publication policy

We will publish our findings open access. We have an affiliation with Tilburg University (TU), which allows us to take full advantage of open access opportunities. Specifically, the

TiU Journal Browser provides access to numerous journals where corresponding authors from Dutch universities and academic hospitals can publish without charge or at a substantial discount thanks to APC arrangements and agreements with Dutch academic publishers.

13. STRUCTURED RISK ANALYSIS

13.1 Potential issues of concern
Not applicable.

13.2 Synthesis
Not applicable.

14. REFERENCES

1. Duff S, Mafilios MS, Bhounsule P, Hasegawa JT. The burden of critical limb ischemia: a review of recent literature. Vasc Health Risk Manag. 2019;15:187-208.
2. McRae PJ, Walker PJ, Peel NM, Hobson D, Parsonson F, Donovan P, et al. Frailty and Geriatric Syndromes in Vascular Surgical Ward Patients. Ann Vasc Surg. 2016;35:9-18.
3. Hughes MJ, Hackney RJ, Lamb PJ, Wigmore SJ, Christopher Deans DA, Skipworth RJE. Prehabilitation Before Major Abdominal Surgery: A Systematic Review and Meta-analysis. World J Surg. 2019;43(7):1661-8.
4. Stacey D, Légaré F, Lewis K, Barry MJ, Bennett CL, Eden KB, et al. Decision aids for people facing health treatment or screening decisions. Cochrane Database Syst Rev. 2017;4(4):Cd001431.
5. Janssen TL, Steyerberg EW, Langenberg JCM, de Lepper C, Wielders D, Seerden TCJ, et al. Multimodal prehabilitation to reduce the incidence of delirium and other adverse events in elderly patients undergoing elective major abdominal surgery: An uncontrolled before-and-after study. PLoS One. 2019;14(6):e0218152.
6. Meulenbroek AL, Steyerberg EW, Janssen TL, van Mil SR, Faes MC, van der Laan L. The Potential Value of Prehabilitation for Preventing Delirium in Elective Surgery for Aneurysms of the Abdominal Aorta. Clin Interv Aging. 2024;19:51-5.
7. Meulenbroek AL, Lanssens G, Fourneau I, Buimer MG, de Groot HGW, Veen EJ, et al. Prehabilitation for delirium prevention in elderly patients with chronic limb threatening ischemia. J Vasc Surg. 2025;81(2):450-8.e7.
8. Rutherford RB, Baker JD, Ernst C, Johnston KW, Porter JM, Ahn S, et al. Recommended standards for reports dealing with lower extremity ischemia: revised version. J Vasc Surg. 1997;26(3):517-38.
9. Mills JL, Sr., Conte MS, Armstrong DG, Pomposelli FB, Schanzer A, Sidawy AN, et al. The Society for Vascular Surgery Lower Extremity Threatened Limb Classification System: risk stratification based on wound, ischemia, and foot infection (WIfI). J Vasc Surg. 2014;59(1):220-34.e1-2.
10. Hardman RL, Jazaeri O, Yi J, Smith M, Gupta R. Overview of classification systems in peripheral artery disease. Semin Intervent Radiol. 2014;31(4):378-88.
11. Jaff MR, White CJ, Hiatt WR, Fowkes GR, Dormandy J, Razavi M, et al. An Update on Methods for Revascularization and Expansion of the TASC Lesion Classification to Include Below-the-Knee Arteries: A Supplement to the Inter-Society Consensus for the Management of Peripheral Arterial Disease (TASC II). Vasc Med. 2015;20(5):465-78.
12. Norgren L, Hiatt WR, Dormandy JA, Nehler MR, Harris KA, Fowkes FG. Inter-Society Consensus for the Management of Peripheral Arterial Disease (TASC II). J Vasc Surg. 2007;45 Suppl S:S5-67.
13. Houghton JSM, Nickinson ATO, Morton AJ, Nduwayo S, Pepper CJ, Rayt HS, et al. Frailty Factors and Outcomes in Vascular Surgery Patients: A Systematic Review and Meta-analysis. Ann Surg. 2020;272(2):266-76.
14. Bellera CA, Rainfray M, Mathoulin-Pélissier S, Mertens C, Delva F, Fonck M, et al. Screening older cancer patients: first evaluation of the G-8 geriatric screening tool. Ann Oncol. 2012;23(8):2166-72.
15. Kempen GI, Brilman EI, Ormel J. [The Mini Mental Status Examination. Normative data and a comparison of a 12-item and 20-item version in a sample survey of community-based elderly]. Tijdschr Gerontol Geriatr. 1995;26(4):163-72.
16. van Asselt DZ, van Bokhorst-de van der Schueren MA, van der Cammen TJ, Disselhorst LG, Janse A, Lonterman-Monasch S, et al. Assessment and treatment of malnutrition in Dutch geriatric practice: consensus through a modified Delphi study. Age Ageing. 2012;41(3):399-404.
