# Supplementary material for: The effect of a multimodal multicomponent Prehabilitation program in Older adults with Chronic limb-threatening Ischemia (POCI-study): A study protocol for a multicenter randomized controlled trial
Source: PLoS One. 2026 Jul 29;21(7):e0354344. doi: 10.1371/journal.pone.0354344 (PMC13419218; doi:10.1371/journal.pone.0354344)
Supplement: S3 File — (DOCX) [file pone.0354344.s003.docx]

# Process evaluation Forms - POCI-study

# **Compliance to the prehabilitation program**

*Patients*

**Please return this form upon hospital admission**

**Evaluation of the Multimodal Multicomponent Prehabilitation Program**

At hospital admission we want to evaluate your compliance to the prehabilitation program. Please complete this questionnaire and take it with you at hospital admission.

**Please answer the following statements:**

**Physiotherapy**

I performed the exercises provided by the physiotherapist at home.

O Yes

O No; because (please provide a reason):……………………………………………………

……………………………………………………………………………………………………………

……………………………………………………………………………………………………………

**Dietician**

I followed the nutritional advice given by the dietician.

O Not applicable

O Yes

O No; because (please provide a reason):……………………………………………………

……………………………………………………………………………………………………………

……………………………………………………………………………………………………………

**Smoking**

I adjusted my smoking habits

Ik heb mijn rookgedrag aangepast

O Not applicable

O Yes, ceased

O Yes, reduced

O No; because (please provide a reason):……………………………………………………

……………………………………………………………………………………………………………

……………………………………………………………………………………………………………

**Alcohol consumption**

I adjusted my alcohol intake in the past few weeks

O Not applicable

O Yes, ceased

O Yes, reduced

O No; because (please provide a reason):……………………………………………………

……………………………………………………………………………………………………………

……………………………………………………………………………………………………………

**Experiences**

Please rate the prehabilitation program on a 0-10 scale (0 means terrible, 10 means excellent). Circle the applicable score.

0 1 2 3 4 5 6 7 8 9 10

Explanation of the provided score: ……………………………………………………………………..

……………………………………………………………………………………………………………

…………………………………………………………………………………………………………………………………………………………………………………………………………………………

#

# **Process evaluation 6 months patients**

**Explanation of the questionnaire:**

- The questionnaire consists of 12 questions
- Carefully read the questions
- Answer the questions to the best of your knowledge
- Mark your answer with a cross
- Give an explanation whenever asked

If necessary, ask your loved ones (informal caregiver, partner, child) to help complete the questions.

**Please provide your name and date of birth:**

Name:

Date of birth:

The questionnaire starts at the next page.

Goodluck completing!

**Question 1: Do you feel the prehabilitation helped you get fitter prior to your treatment?** (circle the applicable score, 1 means ‘did NOT help at all’, 10 means ‘helped A LOT’)

1 2 3 4 5 6 7 8 9 10

**Question 2: How do you think about the prehabilitation program in general?**

…………………………………………………………………………

…………………………………………………………………………

…………………………………………………………………………

…………………………………………………………………………

**Question 3: What mark would you give the prehabilitation program?** (circle the applicable score, 1 means terrible, 10 means excellent)

1 2 3 4 5 6 7 8 9 10

**Question 4: What are positive points of the prehabilitation program?**

………………………………………………………………………………………………

………………………………………………………………………………………………

………………………………………………………………………………………………

………………………………………………………………………………………………

**Question 5: What are negative points of the prehabilitation program?**

………………………………………………………………………………………………..

………………………………………………………………………………………………..

………………………………………………………………………………………………..

………………………………………………………………………………………………..

**Question 6: Did you continue with the exercises provided by the physiotherapist after your operation or percutaneous transluminal angioplasty?**

- Yes, how often

……………………….times a week

.................................times a month

- No, because

…………………………………………………………………………………………………...……………………………………………………………………………………………..........

**Question 7: Did you continue with the nutritional advices provided by the dietician after your operation or percutaneous transluminal angioplasty?**

- Not applicable
- Yes
- No, because

……………………………………………………………………………………….

……………………………………………………………………………………….

In case you continued the nutritional advices, which specific advices did you follow after treatment:

…………………………………………………………………………………………

…………………………………………………………………………………………

…………………………………………………………………………………………

**Question 8: Did you continue to follow any additional recommendations from the prehabilitation program after your treatment?**

- No
- Yes, that is to say

…………………………………………………………………………………………………...

…………………………………………………………………………………………………...

**Question 9: Would you recommend taking part in the prehabilitation program to other patients with complaints of the legs similar to yours?**

- Yes
- No, because

…………………………………………………………………………………………………...

…………………………………………………………………………………………………...

**Question 10: What are your thoughts on taking part in the prehabilitation program together with your informal caregiver?**

……………………………………………………………………………………………………………

……………………………………………………………………………………………………………

……………………………………………………………………………………………………………

……………………………………………………………………………………………………………

**Question 11: What was your most important reason to take part in this study evaluating the effect of prehabilitation?**

……………………………………………………………………………………………………………

……………………………………………………………………………………………………………

……………………………………………………………………………………………………………

……………………………………………………………………………………………………………

**Question 12: Do you have suggestions for the researchers to improve the prehabilitation program for future use?**

……………………………………………………………………………………………………………

……………………………………………………………………………………………………………

……………………………………………………………………………………………………………

……………………………………………………………………………………………………………

**This was the end of the questionnaire. We would like to thank you for your answers and your time!**

# **Process evaluation 6 months informal caregivers**

**Explanation of the questionnaire:**

- The questionnaire consists of 12 questions
- Carefully read the questions
- Answer the questions to the best of your knowledge
- Mark your answer with a cross
- Give an explanation whenever asked

**Please provide your name and date of birth and the name and date of birth of the patient of which you are the primary informal caregiver:**

Name:

Date of birth:

Name patient:

Date of birth patient:

The questionnaire starts at the next page.

Goodluck completing!

**Question 1: Do you feel the prehabilitation helped your loved one getting fitter prior to the treatment?** (circle the applicable score, 1 means ‘did NOT help at all’, 5 means ‘helped A LOT’)

1 2 3 4 5 6 7 8 9 10

**Question 2: How do you think about the prehabilitation program in general?**

…………………………………………………………………………

…………………………………………………………………………

…………………………………………………………………………

…………………………………………………………………………

**Question 3: What mark would you give the prehabilitation program?** (circle the applicable score, 1 means terrible, 10 means excellent)

1 2 3 4 5 6 7 8 9 10

**Question 4: What are positive points of the prehabilitation program?**

………………………………………………………………………………………………

………………………………………………………………………………………………

………………………………………………………………………………………………

………………………………………………………………………………………………

**Question 5: What are negative points of the prehabilitation program?**

………………………………………………………………………………………………..

………………………………………………………………………………………………..

………………………………………………………………………………………………..

………………………………………………………………………………………………..

**Question 6: Did your care recipient continue with the exercises provided by the physiotherapist after the operation or percutaneous transluminal angioplasty?**

- Yes, how often

……………………….times a week

.................................times a month

- No, because

…………………………………………………………………………………………………...……………………………………………………………………………………………..........

**Question 7: Did your care recipient continue with the nutritional advices provided by the dietician after the operation or percutaneous transluminal angioplasty?**

- Not applicable
- Yes
- No, because

……………………………………………………………………………………….

……………………………………………………………………………………….

In case nutritional advices were continued, which specific advices were followed after treatment:

…………………………………………………………………………………………

…………………………………………………………………………………………

…………………………………………………………………………………………

**Question 8: Did your care recipient continue to follow any additional recommendations from the prehabilitation program after the treatment?**

- No
- Yes, that is to say

…………………………………………………………………………………………………...

…………………………………………………………………………………………………...

**Question 9: Would you recommend taking part in the prehabilitation program to other patients with similar complaints of the legs and their informal caregiver?**

- Yes
- No, because

…………………………………………………………………………………………………...

…………………………………………………………………………………………………...

**Question 10: What are your thoughts on taking part in the prehabilitation program together with your care recipient?**

……………………………………………………………………………………………………………

……………………………………………………………………………………………………………

……………………………………………………………………………………………………………

……………………………………………………………………………………………………………

**Question 11: What was your most important reason to take part in this study evaluating the effect of prehabilitation?**

……………………………………………………………………………………………………………

……………………………………………………………………………………………………………

……………………………………………………………………………………………………………

……………………………………………………………………………………………………………

**Question 12: Do you have suggestions for the researchers to improve the prehabilitation program for future use?**

……………………………………………………………………………………………………………

……………………………………………………………………………………………………………

……………………………………………………………………………………………………………

……………………………………………………………………………………………………………

**This was the end of the questionnaire. We would like to thank you for your answers and your time!**
