# Supplementary material for: The effect of a multimodal multicomponent Prehabilitation program in Older adults with Chronic limb-threatening Ischemia (POCI-study): A study protocol for a multicenter randomized controlled trial
Source: PLoS One. 2026 Jul 29;21(7):e0354344. doi: 10.1371/journal.pone.0354344 (PMC13419218; doi:10.1371/journal.pone.0354344)
Supplement: S4 File — (PDF) [file pone.0354344.s004.pdf]

Information brochure prehabilitation outpatient clinic – POCI Study

Preface

Dear Sir/Madam,

You are receiving this information folder because you are experiencing significant symptoms due to a severe narrowing of the arteries in your legs. You will be undergoing treatment for this shortly. You have indicated that you wish to participate in medical-scientific research into prehabilitation. This means that we want to help you prepare as well as possible, both physically and mentally, for your treatment. You have been assigned to group 2: prehabilitation. You will be referred once to the prehabilitation outpatient clinic at your hospital.

When you hear that you need surgery, a lot comes your way. In addition to the emotional experience, you have to arrange all kinds of matters and make decisions. To be able to do this, you need good information.

This folder is not intended to replace verbal information, but to provide you with insight into the steps that will follow in the coming weeks—supplementary to the conversation with the vascular surgeon, the nurse practitioner and/or researcher, and possibly the physiotherapist, dietitian, and geriatrician. You can review everything at your leisure at home.

If you would like to know more about anything, please feel free to ask the nurse specialist or the researchers. They will be happy to provide you with information and can thus contribute to the best possible provision of information or care.

Coordinating physician-researcher

Dr. S. Verbaan Available during office hours — sverbaan@amphia.nl, Tel.nr. 076-5954246

Principal Investigator Amphia Hospital

Prof. Dr. L. van der Laan, vascular surgeon — lvanderlaan@amphia.nl

Principal Investigator Elisabeth Tweesteden Hospital

Present. Dr. P.W.H.E. Friend, vascular surgeon — pwhe.vriens@etz.nl

Principal Investigator Meander Medical Center

Dr. H. Jongsma, vascular surgeon — h.jongsma@meandermc.nl

The team of the Prehabilitation outpatient clinic

Within the specialized prehabilitation outpatient clinic, various specialists work together as a team. This team determines the treatment plan together with you.

Participants include:

- vascular surgeon
- vascular nurse practitioner
- researcher
- physiotherapist
- geriatrician
- dietitian;
- a nurse who assesses with you whether you need extra care after the treatment smoking-cessation-coach

Your visit to the prehabilitation outpatient clinic

Treatment for the severe complaints in your legs and the recovery place a heavy burden on your body. Therefore, it is important to undergo the treatment as fit as possible. Prehabilitation focuses on improving your physical condition and lifestyle. A visit to the prehabilitation outpatient clinic lasts an average of 2 to 4 hours, and we do our best to ensure all appointments to be planned in 1 day.

We kindly ask you to arrive at the outpatient clinic 15 minutes before the start of your appointment. Please bring a recent medication list to your appointment. You can request this from your own pharmacy.

During your visit to the prehabilitation outpatient clinic, you will be guided and screened by a nurse practitioner or researcher. You will receive exercises and advice to ensure you are in the best possible condition for the surgery. It is important that you have a blood test done at the hospital a few days before your appointment.

To improve your general fitness, you can already work on your endurance and strength before the surgery. The physiotherapist will draw up a program with you to improve this. You will find information and examples of exercises further on in this informationfolder

You may be referred to the geriatrician, the dietitian, and/or a smoking cessation coach.

A geriatrician is a medical specialist who focuses on older adults with multiple co-occurring conditions. Through the geriatrician's examination, risk factors for developing delirium are identified and, if possible, treated. In addition, your overall physical and psychological condition is assessed. Over the coming weeks, you will work with the advices at home.

If you have anemia, you will be administered iron intravenously. This will ensure you are stronger going into the surgery.

Based on a few questions and the measurement of your height and weight, your nutritional status is assessed. Good nutrition is important, especially when you are ill. A diet with sufficient energy and protein helps for a faster recovery. You will find tips in the folder that can help you obtain sufficient nutrients. If necessary, you have been given oral nutritional supplements and the dietitian will be consulted to advise you.

Your home situation will be reviewed together with you and your loved ones. This allows us to assess, even before the operation, whether additional care is desired after your admission and during the recovery period. If necessary, we may ask the district nurse to pay you an ‘Advisory Information and Education’ visit prior to the operation.

We advise you to stop smoking. Nicotine use plays a significant role in the development of complications. It impairs blood flow and, consequently, wound healing. Even stopping smoking just two weeks before the operation will reduce these risks. Do you smoke? If so, we can register you for a program in which you quit smoking under supervision. For more information about this program, please consult the ‘quit smoking’ patient leaflet on your own hospital’s website.

We consider it very important to know whether the care you receive from us affects your quality of life. Therefore, we ask you to complete two questionnaires before the operation and three questionnaires after the operation so that we can measure this. Your informal caregiver will also be asked to complete two questionnaires to determine what impact your illness and surgery have on them.

Deciding together

The researchers at the hospital consider it extremely important to tailor the treatment to your personal situation and wishes. We call this process ‘shared decision-making’. On the website of the hospital where you are being treated, you can find an information brochure with videos about shared decision-making.

After your second visit to the vascular surgeon, we will ask you and your caregiver to complete .three questionnaires about shared decision-making

Prehabiliation outpatient clinic checklist

Below, you can check whether you have done everything / have everything with you for your visit to the prehabilitation outpatient clinic:

o Recent medication list (can be obtained from your own pharmacy)
o Blood drawn at the hospital at least 2 days in advance
o Ask a close relative to accompany you to the outpatient clinic
o Please be on time; we ask you to arrive 15 minutes before the appointment

Physiotherapy handbook of prehabilitation

Introduction

You will be undergoing surgery soon. The better your physical condition, mobility, strength, balance, and nutritional status, the faster and better you will recover. Additionally, this reduces the risk of complications after the operation during your hospital stay.

To prepare you as well as possible for the surgery, we offer a training program to optimize your overall fitness, mobility, strength, balance, functionality, and breathing. Naturally, all of this is tailored to your level and within realistic possibilities. In doing so, we are guided by the fact that you yourself can exert a great deal of influence on your own health

Based on physical tests and the information you provide, the physiotherapist gets an idea of your level of fitness. Based on this, you will receive your own training program.

The more you practice at home within your capabilities, the more progress you will make.
If necessary, you will be advised to discuss the program with a physiotherapist in your area and adjust it if needed.

Should you have any further questions, you can always contact the physiotherapist at the hospital where you are being treated. The physiotherapists can be reached at the following telephone numbers:

-
-
-

In this handbook, you will find the exercises, advices, and a review of the information provided.

Information and advice

For adults and the elderly, healthy physical activity is understood to mean the following:

- Engage in at least 150 minutes of moderate-intensity exercise per week, such as walking and cycling, spread over several days. Moving longer, more often, and/or more intensely provides additional health benefits.
- Perform muscle- and bone-strengthening activities at least twice a week, for older adults combined with balance exercises.

This means that a patient must walk for 20 minutes daily, combined with muscle/bone strengthening and/or balance exercises twice a week. Activities can be adjusted during and after hospitalization to get increasingly closer to the physical activity guidelines.

Every extra step you take compared to your normal activity counts.
Try every day, every week, if you can do just a little bit more.
But always taking into account your safety and physical capabilities.
If you have a smartphone, you can download a pedometer with which your effort and progress become clear.

Exercising

It is important to exercise every day. An active lifestyle slows the further development of cardiovascular disease. It even has an improving effect.

Due to your physical condition, it may not be possible to walk or cycle. Try to get moving within your capabilities. Do not sit still too often.

If you enjoy cycling, go for a short ride regularly. This could also be done on a stationary bike, allowing you to cycle briefly several times a day using a light gear.

Tips for exercising:

- Try to stand up for a moment more often. If possible, try to walk short distances
- indoors several times a day. It is better to walk a short distance more often than to walk a long distance once.
- Try to listen carefully to your body.
- Regular light exercise specifically increases blood flow in the legs, among other areas.

The operation

During your hospital stay, we will guide you after the operation in practicing breathing exercises, mobilization, increasing self-reliance, and, if necessary, climbing stairs. Exercises that you can perform at home after the operation will be discussed with you.

We will go through the points of attention relevant to your situation with you.

Walking

It is important to walk at a normal pace for up to 30 minutes every day. You may also break this walking down into shorter sessions, for example, three times ten minutes, six times five minutes, or ten times three minutes.

Did you know that you can improve your overall fitness simply by being more active in your daily life?

Here are a few examples of activities to incorporate into your daily life:

- Walk to the store instead of going by car.
- Walk over to a neighbor for a chat instead of calling.
- Take the stairs instead of the elevator or escalator.
- Get off the bus one stop earlier and walk home.
- When visitors and family arrive, go for a walk with them before tea.
- Garden when the weather permits.
- Stand up when folding the laundry.

In addition to walking for 30 minutes a day, it is also good to keep track of the number of steps you take using a pedometer.

Try to take 100 more steps every day, for example, than the day before.

Tips for walking:

- Try to relax your shoulders and swing your arms gently back and forth.
- Look ahead, not down.
- With every step, the heel lands first, then you push off with your toes.
- Use a walking aid if desired. Safety comes first.
- Enjoy the walk.

Exercises for strength and balance

The exercises on the following pages have a positive influence on balance, endurance, and muscle strength.

Try to do the exercises 3 times a week. After each exercise day, there is always one rest day. The exercises do not all have to be done consecutively; you can spread them out throughout the day or combine them with another activity.

Perform only the checked exercises.

- Perform the exercises slowly: two to three seconds per exercise.
- Perform the exercises 3 sets of 10 repetitions, with both the left and right side, unless otherwise described.
- Rest for one to two minutes between sets.
- Involve family members, informal caregivers, and the immediate social circle.
- Always ensure a safe practice environment

Exercises in bed:

□Gently move both raised knees from the left to to the right, arms spread

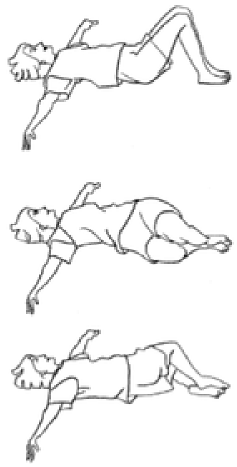

□Lift the fully extended leg off the bed, pull your toes towards you

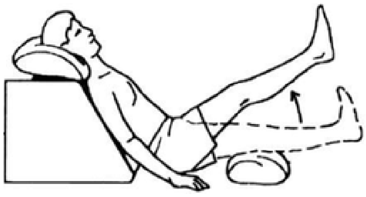

□Turn onto your side as shown, from a seated position ,same way back to supine position: 10x

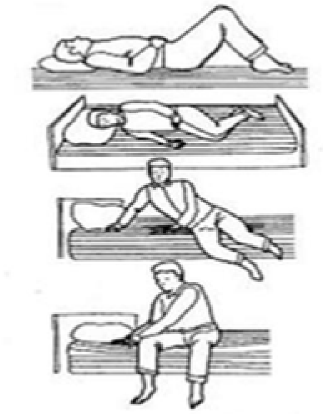

□ From the starting position, push your buttocks off onto the bed: 10x 3 sec

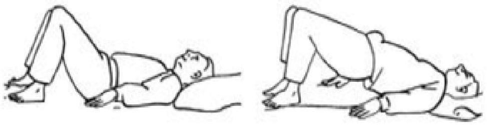

Exercises on and from the chair:

- ◻ Fully straighten your knee: hold for 3 seconds.

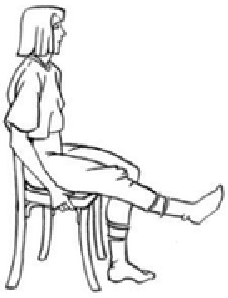

- ◻ Lift your bent knee so that it comes away properly from the chair seat: hold for 3 seconds

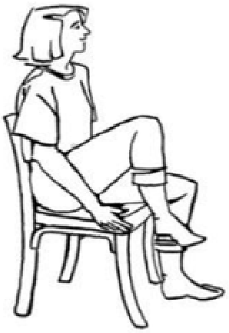

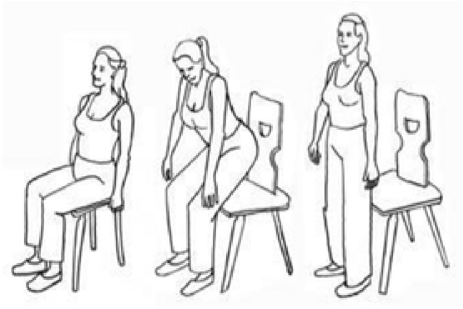

- ◻ Feet shoulder-width apart, slightly slide your buttocks forward and come to a standing position. Make the exercise more challenging by not using your hands or performing the exercise very slowly.

Exercises behind a chair, the kitchen counter, or a firm hold:

□ Place your feet shoulder-width apart,
alternate toes and heels

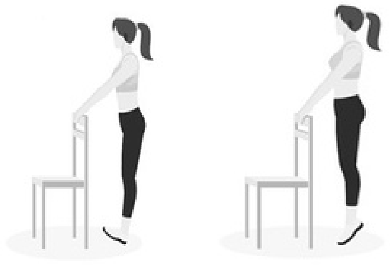

□ Place your feet shoulder-width apart, alternating move your leg sideways

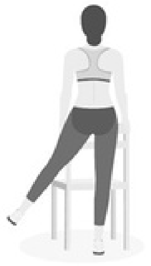

□ Place your feet shoulder-width apart, alternatingly pull up your knee

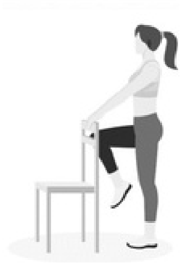

□ Place your feet shoulder-width apart, squat down a bit and straighten up again

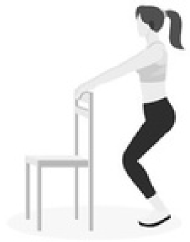

□ Place your feet shoulder-width apart. Try for 60 seconds to stand on one leg. Switch legs

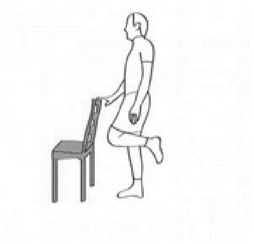

Breathing

After the operation, good ventilation of the lungs—breathing deeply—is very important. This is because some mucus may form in the lungs during the operation and because you may be spending slightly more time in bed than usual. Smoking cessation can also cause a productive cough. It is of great importance that this mucus is coughed up.

Exercise(s) for breathing and/or respiratory muscle training.

Based on a screening, the physiotherapist determines whether it is important for you to do breathing exercises. This may be the case if there is excessive mucus buildup in the lungs or if your respiratory muscles are too weak.

The physiotherapist will let you try any exercises once and determine how long and how often you need to do the exercise daily.

If you become dizzy, unwell, short of breath, or feel unwell, always stop immediately.

Exercise (to be completed by physiotherapist)
Number of exercise sessions per day : 2x daily

Number of minutes/repetitions per exercise session :
Number of minutes of rest :

Advices

Calm breathing

Sit in a relaxed position. Relax your shoulders as much as possible. Breathe in through your nose and breathe out through your mouth. If your nose is blocked, breathe in through your mouth. Breathe out with your lips pressed together slightly, creating a small opening to exhale.

When you inhale, you feel your belly expand. When you exhale, your belly contracts again. Place one hand on your chest and one hand on your belly. This way you can check if you are doing it correctly. Continue breathing like this for two minutes at a calm pace.

Deepen and extend

Breathe in deeply through the nose, not explosively, but as much as you can. Hold your breath for a few seconds. Then calmly exhale the entire contents of your lungs.

Huffing and coughing

These are two ways to remove mucus that may be in the lungs and airways. Huffing is a long, controlled, forceful exhalation. You make a panting sound, think of your glasses or mirror fogging up.

Breathe deeply in through your nose. Open your mouth. Exhale the air calmly with an open throat, not explosively or with maximum force. If you feel the urge to cough, cough thoroughly. Support any wound area by supporting the abdomen with your right hand on your left flank and vice versa, or by placing both hands on the wound. Breathe deeply in through your nose. Cough and apply pressure to the wound area with both hands.

In summary:

- **2 minutes of calm breathing (opening the airways and lungs)**
- **Breathe in deeply 5 times and hold your breath for three seconds.**
- **5x huffing = sighing the air out of the lungs with an open mouth (clearing the mucus from the lungs)**
- **Try to cough up mucus 3 times (get the mucus out of the** airways)
- **Repeat every hour**

Physiotherapy Diary

We would like to ask you to keep track of the days on which you performed the physiotherapist's exercises in the schedule below. If you performed the exercises, circle YES; if you did not, circle NO. If you performed the exercises, please also indicate how often per day you did so. Bring this schedule with you if you are admitted to the hospital.

	**Week 1**	**Week 2**	**Week 3**
**Monday**	Yes / No …….. times per day	Yes / No …….. times per day	Yes / No …….. times per day
**Tuesday**	Yes / No …….. times per day	Yes / No …….. times per day	Yes / No …….. times per day
**Wednesday**	Yes / No …….. times per day	Yes / No …….. times per day	Yes / No …….. times per day
**Thursday**	Yes / No …….. times per day	Yes / No …….. times per day	Yes / No …….. times per day
**Friday**	Yes / No …….. times per day	Yes / No …….. times per day	Yes / No …….. times per day
**Saturday**	Yes / No …….. times per day	Yes / No …….. times per day	Yes / No …….. times per day
**Sunday**	Yes / No …….. times per day	Yes / No …….. times per day	Yes / No …….. times per day

Informationbrochure dietetics Prehabilitation

NUTRITION AND RECOVERY

Energy- and protein-rich snacks and desserts
In preparation for your surgery, it is important that you get enough exercise and eat healthily. This increases the chance of the best possible outcome of the operation and recovery after the operation.

Maintaining a stable weight is important. If you lose weight, you break down muscle mass, which is undesirable. Conversely, if you gain too much weight, you accumulate excess body fat, which is also not favorable for surgery.

You need extra protein before, during, and after the operation for optimal recovery. Protein is an important part of our diet and is used as a building block for the body. Protein is needed, among other things, for the building and maintenance of muscles, but also for wound healing.

,Protein is found primarily in animal products, such as meat (and meat products), fish, chicken ,dairy, eggs, and cheese. However, protein is also supplied by ready-made meat substitutes, nuts peanuts, soy products, and legumes (such as white beans, kidney beans, peas, lentils, and chickpeas). The amount of protein the body needs in this situation is an average of 75-100 grams .of protein per day

It is not always easy to consume so much protein. For this, it may be necessary for you to be temporarily prescribed oral nutrition supplements by a dietitian. You can also increase your .sprotein intake yourself with the following advice

Eat and/or drink daily

- 100 grams of meat, fish, poultry, vegetarian substitute, or legumes
- Generous savory toppings (2-3 slices per slice) (cheese/cold cuts/egg/fish/peanut butter) instead of sweet spread on bread
- 500-600 ml dairy such as milk, soy milk, buttermilk, (drinking) yogurt, custard, quark, skyr
- A handful of unsalted nuts or peanuts
- 3 protein-rich snacks

What kinds of protein-rich snacks can you choose? See the attached list of protein-rich snacks.

When do you eat protein?
The timing of protein intake influences muscle building. Preferably adhere to the following points to get the best effect from the protein:

• Eat at least 4-6 meals per day.

• Ensure that every meal contains 20 to 30 grams of protein (think of a meal with 2 slices of bread with generous toppings and a glass of dairy, or 200 grams of quark with 45 grams of muesli, or an evening meal with at least 75 grams of prepared meat, fish, chicken, poultry, or meat substitute).

• Have another meal with 20 to 30 grams of protein 30 minutes before going to sleep.

• Do you do strength training? Then eat another meal with 20 to 30 grams of protein within 30 to 60 minutes afterward.

In short: eat 20 to 30 grams of protein at breakfast, lunch, dinner, and before bed. Do you do strength training? Then eat an extra meal. Do you want more? Then you can also have snacks.

Are you underweight or do you have unwanted weight loss?

Choose high-calorie, high-protein products as much as possible and eat and drink more often per day (6-8 times), such as full-fat dairy, a cracker with fatty fish, a cube of cheese, or an extra handful of unsalted nuts.

A referral to a dietitian is recommended in this situation. Sometimes, it is also necessary for you to use oral nutritional supplements. These are prescribed by your dietitian.

Are you overweight?

Choose products with few calories and high protein as much as possible, such as low-fat quark, low-fat cheese, low-fat cold cuts, and eggs.

If you do not meet the required protein intake, you can also opt for whey protein when oral nutritional supplements are not necessary. You can add this to water, milk, yogurt, quark, lemonade, or fruit juice. Please note: if you have kidney problems, consult your dietitian first.

You can obtain whey protein at the drugstore. For dosage, consult the packaging or contact the dietitian.

Do you have diabetes?

Then consult your dietitian first. Not all products on this list are suitable for you.

Would you like to know exactly how much protein you eat in a day? Go to the Voedingscentrum's Food Meter via the website https://mijn.voedingscentrum.nl/nl/eetmeter/ or download the Food Meter app. With the Food Meter, you can calculate for yourself how much protein your diet contains.

Questions?

If you have any questions, please contact the dietitian at your hospital. You will find the contact details below:

-

-
-

Energy- and protein-rich snacks and desserts

Sweet	Kcal	Protein
□ Apple turnover	400	4
□ Thick slice of gingerbread with butter and sugar	185	1
□ Egg cake with butter and sugar	220	2
□ Muesli bun or currant bun with butter and sugar	270	4
□ Liège waffle (50 grams)	200	3
□ Pancake (70 grams) with syrup	250	6
□ Slice of butter cake (50 grams)	220	3
□ Stroopwafel (large size)	170	1
□ Filled cookie or apple cake	260	3
□ Liga Milkbreak (1 pack/2 cookies) / Sultana (1 pack)	180	4
□ Kitkat / Mars / Nuts / Twix / Bounty / Snickers	240	4
□ Vifit sport high protein recovery bar (55 grams)	210	20
□ Mini building block (cake pastry, 30 grams frozen product)	100	5.5
□ Eat naturel protein packed reep 45 gram	240	10

Savoury	Kcal	Protein
□ Slice of bread, thick butter and 2 slices of cheese (40 grams)	250	13
□ Sausage roll	310	7
□ Beef salad (140 grams)	254	5
□ Sausage roll	290	9
□ Croquette	150	7
□ Roll of lean ham or other lean cold cuts	25	4
□ 2 tablespoons peanuts/cashews/mixed nuts	260	12
□ Small bag of chips (50 grams)	275	5
□ Cheese cubes (40 grams)	150	10
□ Piece of lean meat (75 grams, cooked weight)	120	18
□ Piece of lean fish (75 grams, cooked weight)	75	18
□ Boiled/fried egg	75	7
□ Smoked salmon (40 grams)	84	9

Drinks

	Kcal	Protein
☐ Cup of coffee with milk and sugar	50	1
☐ Extran orange (energy drink) (200 ml)	120	0
☐ Carton of full-fat chocolate milk (200 ml)	180	7
☐ Carton of full-fat milk (200 ml)	125	7
☐ Carton of grape juice / Taksi / multi-fruit juice / Dubbeldrank	110	0
☐ Melkunie protein shake (225 ml)	170	20
☐ Ready-made or homemade milkshake (200 ml)	150	6
☐ Good Morning breakfast drink (150 ml)	185	5

Examples of energy-rich snacks/desserts Desserts

	Kcal	Protein
☐ Small bowl of full-fat fruit quark (200 ml)	280	10
☐ Small bowl of low-fat quark (200 ml)	120	17
☐ Small bowl of whipped cream custard / indulgent custard (200 ml)	240	5
☐ Small bowl of chocolate mousse (100 ml)	250	3
☐ Small bowl of creamy yogurt + fruit (200 ml)	280	5
☐ Small bowl of Alpro Go on Natural (200 ml) soy-based Cornetto/	135	12
☐ Magnum	280	4
☐ Small bowl of ice cream (2 scoops)	220	3
☐ Small bowl of semolina pudding/ rice pudding with sugar (150 ml)	180	5
☐ Tiramisu, 100 grams	250	3
☐ Skyr yogurt natural or fruit (200 ml)	140	20
☐ Melkunie protein quark (200 ml)	135	20
☐ lactose-free Melkunie protein yogurt (200 ml)	145	20
☐ Melkunie protein pudding (200 ml)	148	20
☐ lactose-free Alpro protein (200 grams)	162	15
☐ Alpro protein pudding (200 grams)	190	20
☐ Lindahls Kvarg (150 ml) (Nestlé)	90	17
☐ Easy-to-eat (pudding 55 grams, frozen product)	136	8
☐ Ehrmann high protein pudding (200 ml)	150	20
☐ Koupe high-protein ice cream (100 g)	160	12

- Protein powder from various brands based on whey protein, or for vegans for example, based on peas. This can be incorporated into water, milk, yogurt, quark, lemonade, fruit juice, smoothies, etc.

1 scoop provides: 15-20 grams of protein (see packaging).

For more ideas, consult the database of the Malnutrition Steering Group at De-Eiwitwijzer.pdf

Protein-rich alternatives
To order energy and protein-rich products online, such as Bouwsteentjes, Easy to eat, or a protein-rich milkshake, go to the website: www.boostbuddies.com

Carezzo makes everyday products for people who need extra protein in a tasty and flavorful way. This includes bread, fruit drinks, soup, ice cream, and light meals. Most products are fortified with 10 grams of protein per serving.

Nutritional declaration (nutritional label)
The range of potentially suitable products varies by supermarket and is constantly subject to change. In addition, the composition of a similar product (for example, creamy yogurt) can also differ by brand or flavor variant.

To ensure that a product contains sufficient energy and protein, it is important to check the nutritional information on the packaging. See the example of a nutritional information on the right. Desserts containing at least 125 kcal and at least 3 grams of protein per 100 grams or 100 ml are suitable.

Gemiddelde voedingswaarden

	Per 100 g	1 portie (65g)
**Energie**	810kJ/194 kcal	526kJ/126 kcal
**Vet**	13,3 g	8,6 g
waarvan		
verzadigd vet	3,0 g	2,0 g
enkelvoudig onverz. vet	5,1 g	3,3 g
meervoudig onverz. vet	1,8 g	1,2 g
**Koolhydraten**	0,5 g	0,3 g
waarvan		
suikers	0,4 g	0,3 g
polyolen	0 g	0 g
zetmeel	0 g	0 g
**Vezels**	1,6 g	1,0 g
**Eiwitten**	18,5 g	12,0 g
**Zout**	1,95 g	1,25 g

Source:
www.voedingscentrum.nl

Explanation for filling out the food diary (found in Appendix E4. Food Diary)

- The purpose of the food diary is to gain a clear picture of your daily diet. The dietitian can calculate your nutritional intake based on this food diary.
- Immediately record everything that has been eaten and drunk. Do not save filling it out until the evening, as there is a very good chance you will forget something.
- Write everything down, including small snacks such as candy, chips, etc. Don't forget the drinks, such as tea, coffee, water, and fruit juice.
- Describe exactly what you are eating, including the brand name if applicable, for example:
    - Butter: butter / Becel diet margarine / margarine
    - Cold cuts: smoked meat / tongue sausage / cooked ham
    - Cheese: 48+ / Milner 30+ / Slankie spreadable cheese
    - Bread: wholemeal / brown / white bread
- Note down the quantities of the food items eaten as much as possible, such as: a cup of milk, a slice of bread, a teaspoon/lump of sugar, 3 small boiled potatoes, 100 grams of raw meat fried in olive oil (2 tablespoons), 3 serving spoons of green beans with a knob of butter, a handful of peanuts, a small bag of chips, etc.
- Measure the capacity of the glasses, cups, mugs, bowls, etc. used (using a measuring cup). For example:
  Coffee cup = ……….ml
  Cup = ……….ml
  Mug = ……….ml
  Glass = …….ml
  Small bowl = ……..ml
- Under preparation, indicate whether and how the food item was prepared: for example, boiled or fried.
- Under additions, indicate whether anything; for example, salt, herbs, mayonnaise, has been added to a food item.
- Particulars are, for example, the food item was prepared by someone else (it is not exactly clear how it was prepared), eaten at a party, stomach ache so not eaten or ate less.

Good luck filling it out!

Home Situation and Informal Care

Go home with confidence after your hospital stay
You will be admitted to the hospital soon. At some point, your treatment in the hospital will be over and you can return home. Before you go home, it is important that a number of matters are discussed with you.

Do you know, for example, what you can do if you are still in pain at home? Or in which situations you should contact a doctor and who you can reach for this? Is it clear to you which activities you can already do at home and which you cannot yet?

This checklist contains the most common questions patients have when they go home. You can use this during your discharge interview or during other conversations you have with a nurse or doctor about the period after your admission.

If your nurse or doctor wishes to discuss matters regarding your discharge with you, you may consider having a trusted person present during the conversation. Two people hear more than one.

Please bear in mind that a dismissal interview does not always clearly take place at a single moment. A number of points on this checklist must already be clear well before the moment of dismissal.

Checklist for you and your loved ones
Daily activities

- √ Do you know what you can already do yourself at home and what you are (for the time being) advised against?

  You can think of bathing or showering, household activities, driving, and the like.

Aftercare

- √ Is it clear to you whether you will still need extra care and assistance when you are back home?
- √ If so, is it clear what you will need (temporary) care and assistance with?
- √ What care and support can your loved ones provide you at home?
- √ Who else could you call upon? For example, neighbors, home care, volunteers or homecare
- √ Do you know where you can go for help and information?

Aids

- √ Have you received information about assistive devices you need at home?
- √ Have you received clear instructions on the use of these aids?
- √ Have aids and/or adaptations that you need immediately after your discharge been organized?
- √ Do you know which wound dressing materials you need at home, and do you have a prescription for them?

Consequences for your daily life

- √ Do you know what the consequences of the illness and the hospitalization can be?
- √ Is it clear to you and your informal caregiver what discomforts you may encounter in your daily life?
- √ Is it clear to you what you can do yourself to reduce your symptoms and promote recovery?

Medication

- √ Have you received sufficient explanation from the specialist regarding the use of medication?
- √ If you need to take multiple medications, do you have sufficient information about combining these medications?
- √ Do you know which side effects may occur?
- √ Do you need recipes for your home situation?
- √ Do you need medication for your first day at home? The pharmacy does not always have medicine in stock.

Follow-up appointments

- √ Have follow-up appointments been made with the specialist?
- √ Do you have information about how to reach the department, by phone or email?
- √ Are there follow-up appointments with other healthcare providers in the hospital?
- √ Are they reachable by phone or email?

Other specialists

- √ Are there other specialists you are being treated by (including those in other hospitals) informed about your admission and medication?

Support

- √ Do you or your linformal caregiver need support from others after your discharge?
- √ Do you know where you can seek that support?
- √ Have you received information about patient associations? You can obtain this information from the Patient Service Bureau.

This checklist was prepared by Zorgbelang Brabant© www.zorgbelang-brabant.nl

Informal care

Caring for a loved one can be very intensive, which is why you, as an informal caregiver, need time off. We call this time off respite care. With this, professionals or volunteers (temporarily) take over your care duties.

Information about this care can be found at:

https://www.rijksoverheid.nl/onderwerpen/mantelzorg/vraag-en-antwoord/hoe-kan-ik-als-mantelzorger-hulp-bij-de-zorg-krijgen

Mezzo is the national association for everyone who cares for a loved one: https://www.mezzo.nl/

As an informal caregiver, you can contact Mezzo's 'Informal Care Line' with

questions
